# Epithelial to Mesenchymal transition, eIF2α phosphorylation and Hsp70 expression enable greater tolerance in A549 cells to TiO_2_ over ZnO nanoparticles

**DOI:** 10.1038/s41598-018-36716-2

**Published:** 2019-01-24

**Authors:** Ansie Martin, Angshuman Sarkar

**Affiliations:** CMBL, Department of Biological Sciences, Birla Institute of Technology and Sciences, K K Birla Goa Campus, Sancoale, South Goa 403726 India

## Abstract

Type II alveolar cells are highly robust in nature, yet susceptible to aerosolized nanoparticles (NPs). Dysfunction in these specialized cells, can often lead to emphysema, edema, and pulmonary inflammation. Long-time exposure can also lead to dangerous epigenetic modifications and cancer. Among the manufactured nanomaterials, metal oxide nanoparticles are widely encountered owing to their wide range of applications. Scores of published literatures affirm ZnO NPs are more toxic to human alveolar cells than TiO_2_. However, signalling cascades deducing differences in human alveolar responses to their exposure is not well documented. With A549 cells, we have demonstrated that epithelial to mesenchymal transition and an increased duration of phosphorylation of eIF2α are crucial mechanisms routing better tolerance to TiO_2_ NP treatment over exposure to ZnO. The increased migratory capacity may help cells escape away from the zone of stress. Further, expression of chaperone such as Hsp70 is also enhanced during the same dose-time investigations. This is the first report of its kind. These novel findings could be successfully developed in the future to design relief strategies to alleviate metal oxide nanoparticle mediated stress.

## Introduction

With the advent of synthesized nano-materials particularly metal oxide (MeOx) nanoparticles (NPs)^1^, human exposure becomes inevitable and nanotoxicology research is now gaining attention. High presence of MeOx NPs at sites surrounding factories as compared to clean areas^2^ has been correlated with increase in pulmonary diseases including exacerbation of bronchial asthma^3,4^. Thus, there is a growing need for elucidation of the cytotoxic effects of MeOx NPs on human respiratory health^5^. Many studies have been carried out in the past decades towards this endeavour. We have already presented a concise report on the current knowledge in the field so far, through Martin and Sarkar^5^. The trend of decreasing toxicity among the most widely applied nanomaterials follows; CuO > ZnO > Co_3_O_4_ ~ Sb_2_O_3_ > Mn_3_O_4_ > Al_2_O_3_ > TiO_2_ > Fe_2_O_3_. Aspect ratio of nanomaterials, chemical identity and protein corona interactions dictate the level of toxicity. The level of dissolution after uptake is more significant than just internalization. Different cellular modes of death are encountered with nanotoxicity ranging from apoptosis, necrosis to autophagy. DNA damage, structural changes, modulation in gene and protein expression, are all a common consequence of nanoparticle exposure^5^.

There is however, insufficient information available over what precise signalling cascades may contribute to alleviation of cellular stress and recovery of vital functions from nanotoxicity. In addressing this research lacunae, we have conceptualized a comparison-based study of the differences in cellular responses constituting exposure between less lethal and highly lethal nanoparticles. We have chosen two candidate metal oxide nanoparticles of different toxicities to alveolar A549 cells (Adenocarcinomic human alveolar basal epithelial cells, type II); ZnO (Zinc oxide) and TiO_2_ (Titanium dioxide), ZnO being more lethal than TiO_2_ ^6^. Further it is also widely documented that higher toxicity of ZnO treatment with respect to TiO_2_, manifests in higher cell lethality, DNA damage and cellular lesions^7^.

We have organized our study by evaluating viability first and foremost. This investigation would substantiate the existing knowledge from literature on the degree of lethality between ZnO and TiO_2_ treatment. Changes in the cellular morphology was evaluated thereafter. It is one of the first responses to any change in cellular environment and a key indicator of cellular stress^8^. Changes in cell and nuclear morphology as a dose and time dependent function of nanoparticle exposure was studied through Hoechst staining and characterized by the expression of Rho family members of Small GTPases primarily Rac, Rho and cdc42^9^. These proteins are widely known to regulate cytoskeletal organization^10^. They cycle between an inactive (GDP-bound) and an active (GTP-bound) conformation in which they interact with specific effector proteins^11^. Activation of Rho promotes the formation of stress fibers and focal adhesion complexes^12^, Rac promotes the polymerization of actin at the cell membrane, producing lamellipodia and membrane ruffles^13^ and cdc42 promotes the formation of filopodia and microspikes at the cell periphery^14,15^. Thus, expression of Rho family of Small GTPases (Rac1, RhoA and cdc42) were evaluated both at mRNA and protein level to assess any differences in morphology attributed by changes in their expression.

Changes in protein expression owing to MeOx exposure was analyzed next by studying the inhibition of global protein synthesis, characterized by the phosphorylation of eIF2α^16^. The eukaryotic initiation factor (eIF2) is a well-known translation factor, and its phosphorylation is one of the first events to occur during Integrated Stress responses^17^. eIF2 is a multimeric protein consisting of 3 subunits; α, β and γ. Their sequences are greatly sustained across several species indicating possible roles crucial to cellular viability^18^. The eIF2α phosphorylation at Ser51 is also a highly conserved and adaptive response that can cause down regulation of translation initiation under several types of stresses and regulate gene expression^16^. It also routes in unfolded protein responses through PERK; PKR like endoplasmic reticulum kinase^19^. Human eIF2α accepts phosphate groups from kinases PKR (Double stranded RNA activated protein kinase); activated in response to viral infection^20^ and interferons in mammalian cells^21^. Also, the expression of HRI (Heme regulated inhibitor of translation); activated in response to heme deprivation, heavy metals^22,23^ and GCN2 in response to nutrient deprivation^24^ results in phosphorylation of eIF2α at residue 51-serine. Phosphorylation of eIF2α was hence studied as a dose and time dependent function of MeOx NP toxicity to quantify the level of Integrated stress response.

As ER stress increases with increased accumulation of unfolded proteins, transcription factors ATF6, XBP1, ATF4 and ATF 5 are sequentially activated^17^. This is triggered by GRP78/Bip (78KDa glucose regulated protein or binding immunoglobulin protein) dissociation from the ER domains of ATF6, IRE-1 and PERK respectively, activating them in the process^25^. GRP78 or Hsp70 is a stress related chaperone which is crucial for activation of all ER transmembrane signalling molecules^26^ and may also be expressed while eIF2α stays phosphorylated through activation^27^. Thus, studying the expression of Hsp70 enables analysis of the degree of unfolded protein response triggered to MeOx NP toxicity. To better understand such cellular responses, Hsp70 expression is evaluated at the protein level, for both ZnO and TiO_2_ exposure on A549 cells.

Additionally, for further validation of some of our results, invasion assay was performed to test migratory potential and wound healing assay was carried out to evaluate proliferation capabilities. Cell movement is ordained by a series of signal transduction pathways that include small GTPases, cytoskeleton-modifying proteins, kinases, lipid secondary messengers and motor proteins. Cells achieve movement when different signalling cascades are consistently presented in specific locations within the cell while maintaining potency of response to extra cellular triggers. Both epithelial and mesenchymal cells can migrate, however, mesenchymal phenotype has increased invasive capabilities, combined with a greater resistance to cell death^28^. Further since, increased cellular migration is often a consequence to epithelial to mesenchymal transition^29^, therefore, MeOx NP treated samples were also tested for it.

## Results

Where ever required, pictures were post processed for background reduction and contrast enhancement using PhotoScape, MOOII TECH, Korea. Analysis was performed by ImageJ, NIH. Standard deviation from 3 independent experiments has been annotated.

### The trypan blue dye exclusion test

**Figure 1:**
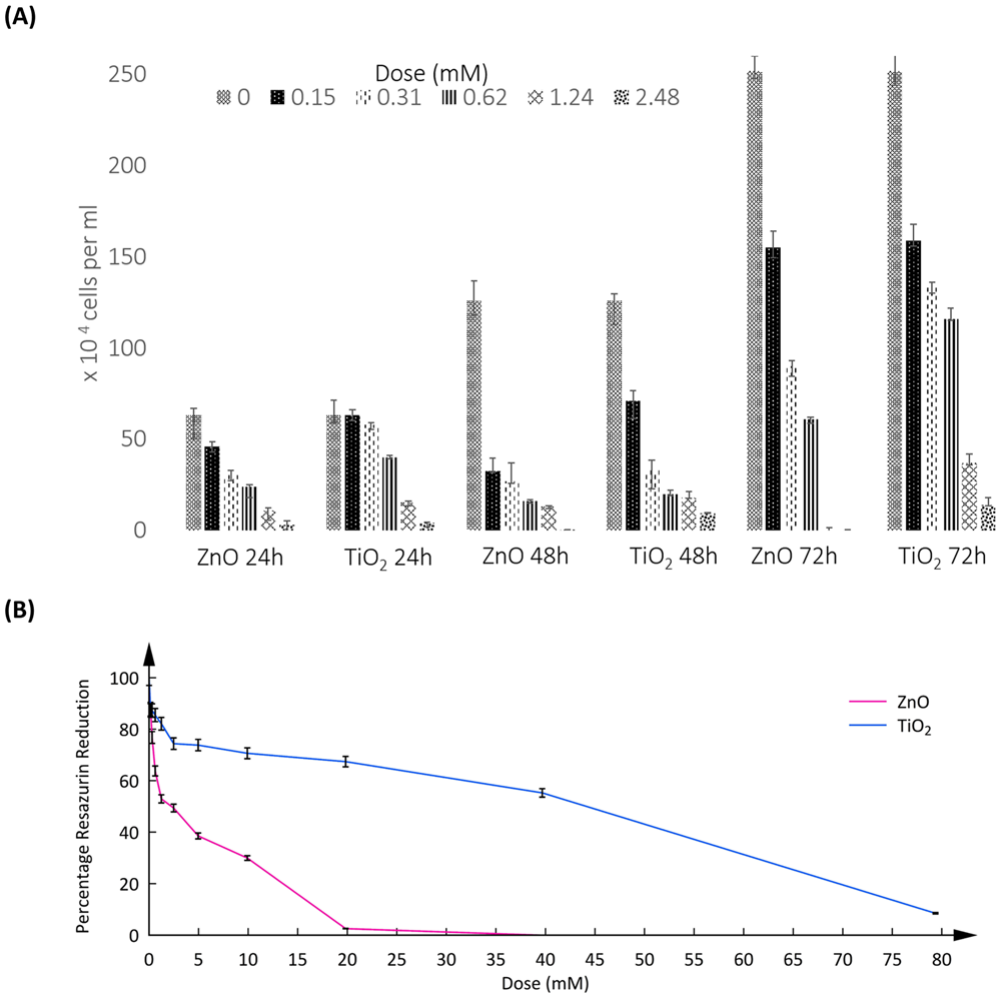
(**A**) Viability assay by trypan blue dye exclusion method. Unstained cells were counted by Neubauer’s haemocytometer and plotted as a function of dose and incubation time to TiO_2_ and ZnO NP treatment. P values are as follows; ZnO 24h- 0.002953 (**), TiO_2_ 24h- 0.000267 (***), ZnO 48h- 0.05 (*), TiO_2_ 48h- 0.023576 (*), ZnO 72h- 0.013468 (*) and TiO_2_ 72h- 0.002275 (**). Average values are plotted with standard deviation indicated through error bars for 3 independent experiments. (**B**) Assessment of mitochondrial dysfunction. Percentage resazurin reduction is plotted against dose of exposure for TiO_2_ and ZnO NP treatment. Resazurin reduction value of the untreated control is considered as a positive control with 100% reduction. P values are as follows; ZnO- 0.000074 (***) and TiO_2_- 6.48E-11 (***). Average values are plotted with standard deviation indicated through error bars for 3 independent experiments.

ZnO exposed cells documented a percentage viability of 73, 48, 38, 17 and 5 for respective doses (in mM) of 0.15, 0.31, 0.62, 1.24 and 2.48 at 24 hours of incubation (Fig. 1A). At 48 hours, the viability percentages dropped to 26, 21, 13, 10 and 0 respectively. Cells recovered slightly at 72 hours until an exposure dose of 0.62 mM, while at higher doses the cell death increased. Viability data at 72 hours for doses (in mM); 0.15, 0.31, 0.62, 1.24 and 2.48 were 62, 36, 24, 0.2 and 0% respectively.

A similar pattern of viability response is seen in cells exposed to TiO_2_ but with less cell death. At 24 hours of incubation, for doses (in mM) 0.15, 0.31, 0.62, 1.24 and 2.48 the viability percentages were 99.6, 92, 64, 24 and 7. The cell death dropped with further incubation unto 48 hours with percentage viability documented at 57, 26.2, 16, 14.3 and 8 correspondingly. Again, cell recovery was seen with further incubation at 72 hours for doses up till 0.62 mM. Viability percentages at 72 hours registered at 63, 53, 46, 15 and 6 for the evaluated set of doses.

### Resazurin Reduction assay

The resazurin reduction percentage (Fig. 1B) for ZnO at doses (in mM) 0, 0.15, 0.31, 0.62, 1.24, 2.48, 4.96, 9.92, 19.84, 39.68 and 79.36 (I to XI) were 79.7, 79.1, 73.2, 71.8, 67.8, 60.2, 43.9, 6, 1.9 and 0 respectively. Corresponding values for TiO_2_ exposure were 87.3, 87.3, 85.5, 82.1, 74.4, 73.8, 70.7, 67.4, 55.3 and 8.5. LD_50_ for ZnO exposure was 2.26 mM while for TiO_2_ was 44.15 mM.

### Morphological Documentation

**Figure 2:**
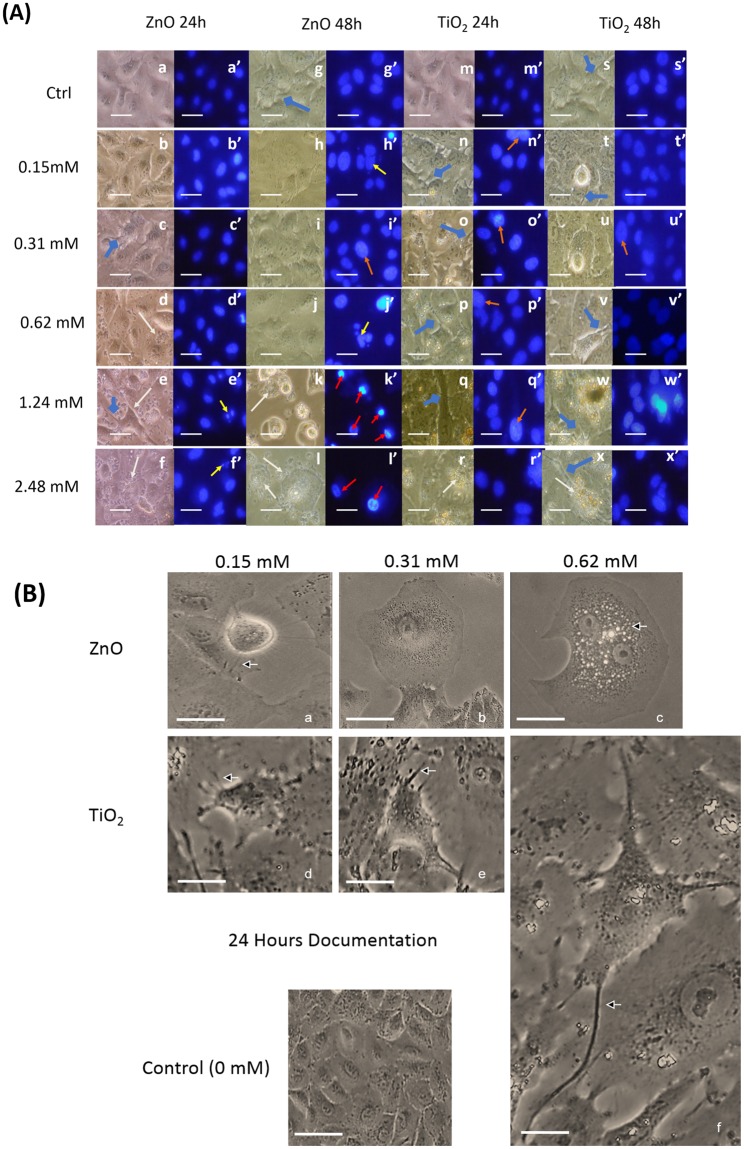
(**A**) Dose and time dependent morphological documentation of MeOx NP treatment by Hoechst Staining. Inverted microscope pictures of A549 cells treated with nanoparticles (a,b etc.) are laid adjacent to Hoechst pictures (a’,b’ etc.) of the same field. Scale bar: 40 µm. The comparative documentation enables in assessment of changes with nuclear morphology as a dose and time dependent exposure of nanoparticles. (**B**) Dose dependent morphological documentation of a single cell to MeOx NP treatment. Changes in cell structure to MeOx NP treatment, for 24 hours at dose points of 0.15–0.62 mM is photographed using an inverted microscope and compared to the untreated control. Scale bar: 20 µm. ZnO treatment results in increased vacuole like structures, while TiO_2_ treatment remarkably extends filopodia.

Enlargement of nucleus is often an indicator of cellular activity involving regulation of gene expression and chromatin organization^30^. Hoechst staining (Fig. 2A) showed enlargement of nucleus (such as in i’, n’, o’, p’, q’ and u’ shown by orange arrows) and necrotic like cells more than apoptotic cells with increasing exposure to ZnO NPs. In comparison, response to TiO_2_ NPs showed a reduction in cell number only after exposure to 50 µg/ml (0.62 mM) TiO_2_. Necrotic like cells with ruptured plasma membrane^31^ (indicated by arrows; d, e, f, k, l, r and x) were seen starting from 200 µg/ml (2.48 mM) for TiO_2_ NP exposure and from 50 µg/ml (0.62 mM) for ZnO. Few apoptotic cells, characterized by cellular blebbing^32^ were also visible in cells exposed to ZnO NPs (represented by yellow arrows in h’, j’, e’ and f’). Nuclei characterized by increased fluorescence were commonly seen in cells exposed to ZnO NPs starting from 1.24 mM (as depicted by red arrows in k’ and l’). Under constant Hoechst dose and UV exposure, increase in fluorescence might indicate a change in the structure of nucleus. Spherical morphology was seen in some TiO_2_ exposed cells (blue arrows in t and u). This morphology is usually associated with detachment from basal lamina and often presents a lack of anchorage dependent growth^33^. Filopodial spikes often present in migrating cells^34^ were seen more in response to TiO_2_ treatment than to ZnO (highlighted by blue diamond arrows in c, e and g for ZnO treated samples and n, o, p, q, s, t, u, v, w and x in TiO_2_ exposed cells).

Figure 2B demonstrates the differences in morphological changes of a single cell following ZnO and TiO_2_ exposure at 24 hours. The panel for ZnO is already published in Santimano *et al*.^35^. Morphological alterations for ZnO from 24 hours to 72 hours follows a dose dependent pattern. At 0.15 mM of exposure, stress fibres are visible in a cell enlarged as compared to the control (Fig. 2Ba), upon 0.31 mM (Fig. 2Bb) of treatment cells appear more flattened. Further stress up till 0.62 mM (Fig. 2Bc) is presented with spicules and vacuole like granules that seem to fill the cytoplasmic periphery. In the case of morphological response to TiO_2_ NPs, Fig. 2Bd–f, we find increased number of elongated cell protoplasm as indicated by the arrows. These extensions are most likely filopodial spikes.

### mRNA level expression of Small GTPase

**Figure 3:**
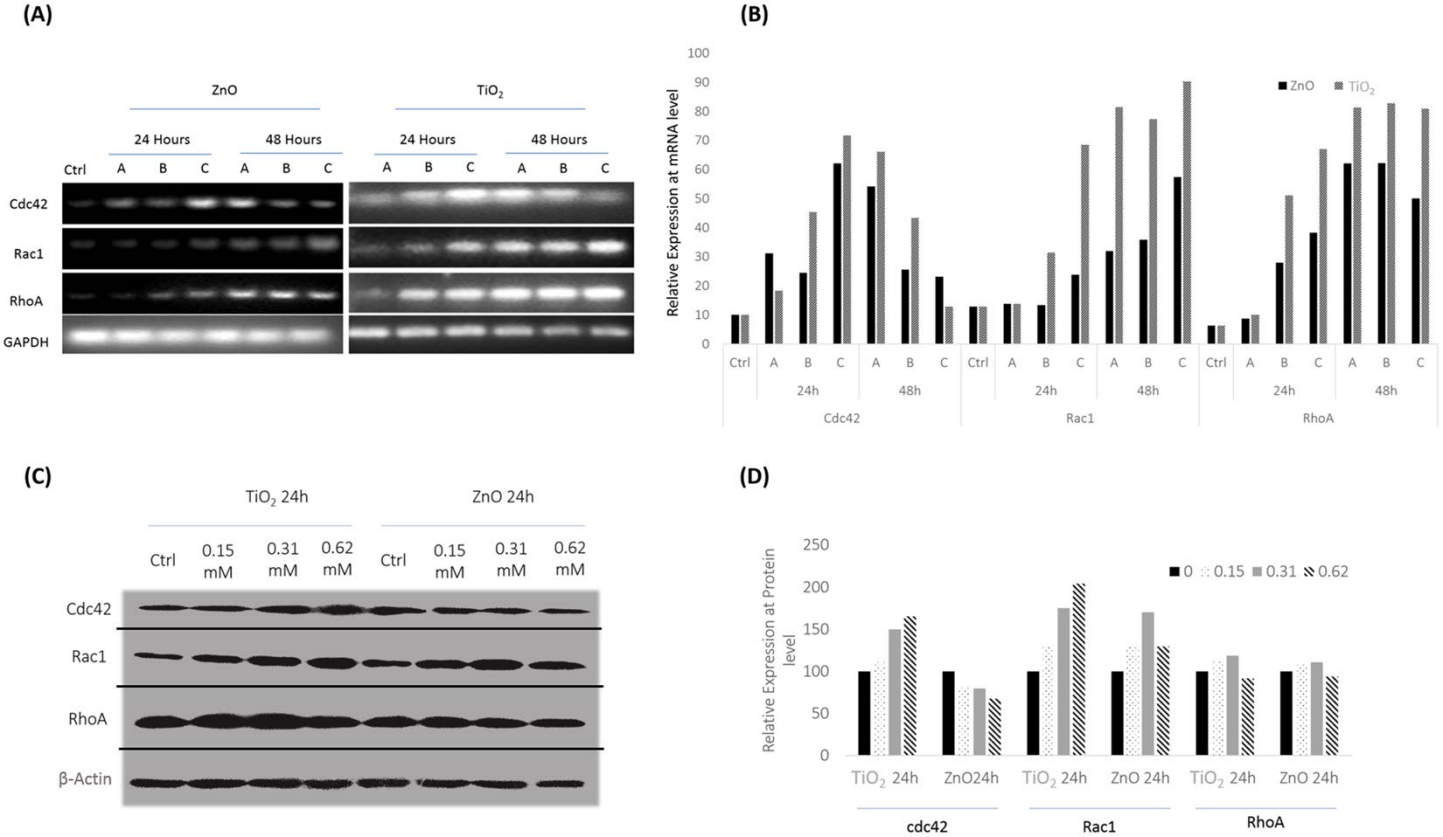
(**A**) Small GTPase expression at mRNA level by RT PCR. 24 hours expression at mRNA level of Small GTPase members; cdc42, Rac1 and RhoA in a dose dependent manner are recorded by photographing with a Gel Documentation set up. Doses: A-0.15, B-0.31 and C- 0.62 mM. The best gel of 3 independent experiments is shown here. Results of the independent experiments were following a similar trend in expression. (**B**) Statistical analysis of Small GTPase expression at mRNA level. Expression profile of the best representative gel is plotted for cdc42, Rac1 and RhoA, relative to the internal control GAPDH in response to MeOx NP treatment. P values are as follows for ZnO NP treatment; cdc42 24h- 0.0268 (*), cdc42 48h- 0.02424 (*), Rac1 24h- 0.04052 (*), Rac1 48h- 0.04892 (*), RhoA 24h- 0.043598 (*) and RhoA 48h- 0.027501 (*). TiO_2_ exposure resulted in P values of; cdc42 24h- 0.01366 (*), cdc42 48h- 0.04172 (*), Rac1 24h- 0.028929 (*), Rac1 48h- 0.02282 (*), RhoA 24h- 0.050 (*) and RhoA 48h- 0.044773 (*). (**C**) Small GTPase expression at protein level by western blot analysis. 24 hours expression pattern at the protein level, of Small GTPase members; cdc42, Rac1 and RhoA is recorded in a dose dependent manner. The best blot of 3 independent experiments is shown here. Results of the independent experiments were following a similar trend in expression. (**D**) Statistical Analysis of Small GTPase expression at protein level. Expression profile of best representative blot is plotted for cdc42, Rac1 and RhoA, relative to the internal control Beta Actin in response to MeOx NP treatment. P values for ZnO treatment at 24 h are; cdc42–0.001917 (**), Rac1- 0.03973 (*) and RhoA- 0.00398 (**). TiO_2_ exposure for 24 h resulted in P values of; cdc42- 0.007685 (**), Rac1- 0.0097452 (**) and RhoA- 0.008882 (**).

cdc42 increases at 0 up to a dose of 0.62-mM at 24 hours and decreases from 0.15 to 0.62-mM at 48 hours for both TiO_2_ and ZnO exposure (Fig. 3A). Our experimental evaluation records an average of 10–20% more expression of cdc42 with TiO_2_ NPs exposure as compared to ZnO. Rac1 increases at exposure from 0.15 to 0.62-mM for both 24 hours and 48 hours to ZnO and TiO_2_ exposure. At 48 hours, Rac1 shows 30–40% more expression at the mRNA level in TiO_2_ treated cells against ZnO NPs. RhoA also increases in a dose and time dependent manner in response to both ZnO and TiO_2_. Although at 0.62 mM for 48 hours it begins to reduce for ZnO. RhoA expression to TiO_2_ stays elevated around 60% more than control from doses 0.15–0.62 mM. Densitometric analysis is represented in Fig. 3B.

### Protein level expression of small GTPase

cdc42 increases up to the evaluated dose of 0.62 mM for TiO_2_ while it down regulates for ZnO at 24 h (Fig. 3C). At the highest dose evaluated; 0.62 mM, cdc42 expression increased 60% of control in TiO_2_ exposed cells, while decreased 40% to ZnO treatment. Rac1 remains increased in expression from 0.15 to 0.62-mM in response TiO_2_ while, ZnO exposed cells see upregulation till 0.31 mM and the expression is downregulated thereafter at 0.62 mM. Highest expression was noted at 0.62 mM for TiO_2_ with 100% more than control, while the peak for ZnO was observed at 0.31 mM at 50% more than untreated samples. RhoA does not show any drastic increase in expression pattern, although the up regulation is seen more pronounced in TiO_2_ exposure than ZnO. Highest expression of RhoA was seen at 0.31 mM in cases of both ZnO and TiO_2_ treatment, again TiO_2_ dictated 10% more expression than ZnO treated samples. Densitometric analysis is presented in Fig. 3D.

### Phosphorylation status of eIF2α

**Figure 4:**
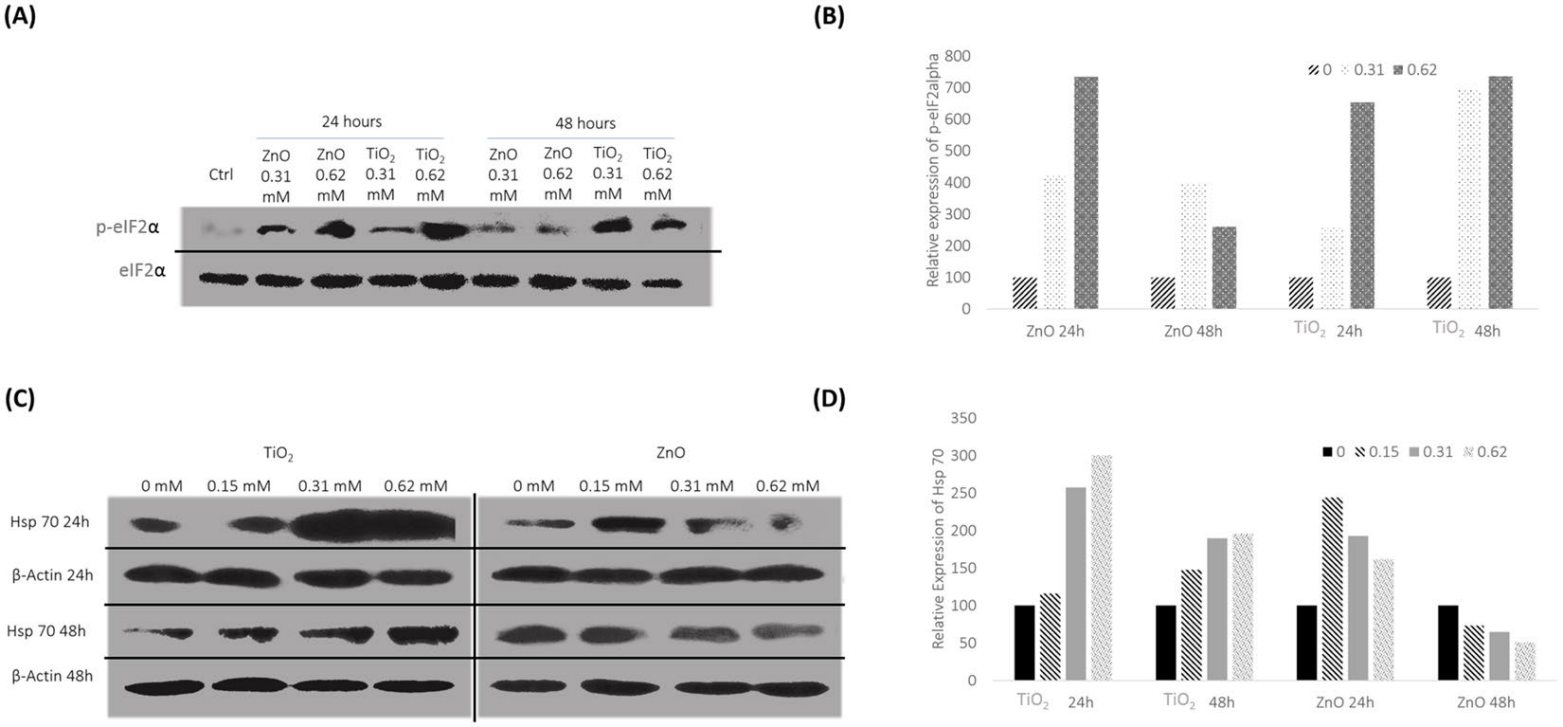
(**A**) Evaluation of the phosphorylation status of eIF2α by western blot analysis. Dose and time dependent analysis of the phosphorylation status of eIF2α was carried out as documented. The best blot of 3 independent experiments is shown here. Results of the independent experiments were following a similar trend in expression. (**B**) Statistical Analysis of the phosphorylation of eIF2α. Expression profile of p-eIF2α from the best representative blot is plotted, after normalizing with the internal control; the total eIF2α following MeOx NP treatment. P value were; ZnO 24h- 0.044069 (*), ZnO 48h- 0.04796 (*), TiO_2_ 24h- 0.0428075 (*) and TiO_2_ 48h- 0.05002 (*). (**C**) Analysis of the Hsp70 expression at the protein level by western blot analysis. Dose and Time dependent expression of Hsp 70 was recorded as shown. The best blot of 3 independent experiments is shown here. Results of the independent experiments were following a similar trend in expression. (**D**) Statistical Analysis of Hsp70 expression. Dose and time dependent expression of Hsp70 is plotted from the best representative blot, after normalizing with the internal control; Beta Actin in response to MeOx treatment. P values are; TiO_2_ 24h- 0.010503 (*), TiO_2_ 48h- 0.024978 (*), ZnO 24h- 0.04993 (*) and ZnO 48h- 0.0046068 (**).

p-eIF2α expression increased up to the evaluated 0.62 mM for both ZnO and TiO_2_ expression at 24 hours (Fig. 4A). With further incubation, up till 48 hours however, p-eIF2α levels dropped by 400% at 0.62 mM in case of ZnO exposure but stayed elevated in response to TiO_2_ exposure. Densitometric analysis is given in Fig. 4B.

### Hsp70 expression

With TiO_2_ exposure at 24 hours, Hsp70 expression upregulated with increase in dose up till the documented dose of 0.62 mM at 300% of control expression (Fig. 4C). Further incubation of 48 hours, also showed an increase in Hsp70 expression with increase in dose, though maxima reached among the documented doses was at 0.62 mM with 196% of control expression.

However, ZnO treatment for 24 hours reached maxima at 244% of control at 0.15 mM. There after further increase in dose, observed a decrease in Hsp70 expression. Increased incubation of 48 hours documented downregulation of Hsp70 with expression percentage at 100, 73, 64 and 50.5 for doses 0, 0.15, 0.31 and 0.62 mM respectively. Densitometric analysis is plotted in Fig. 4D.

### mRNA level expression analysis for EMT (epithelial to mesenchymal transition)

**Figure 5:**
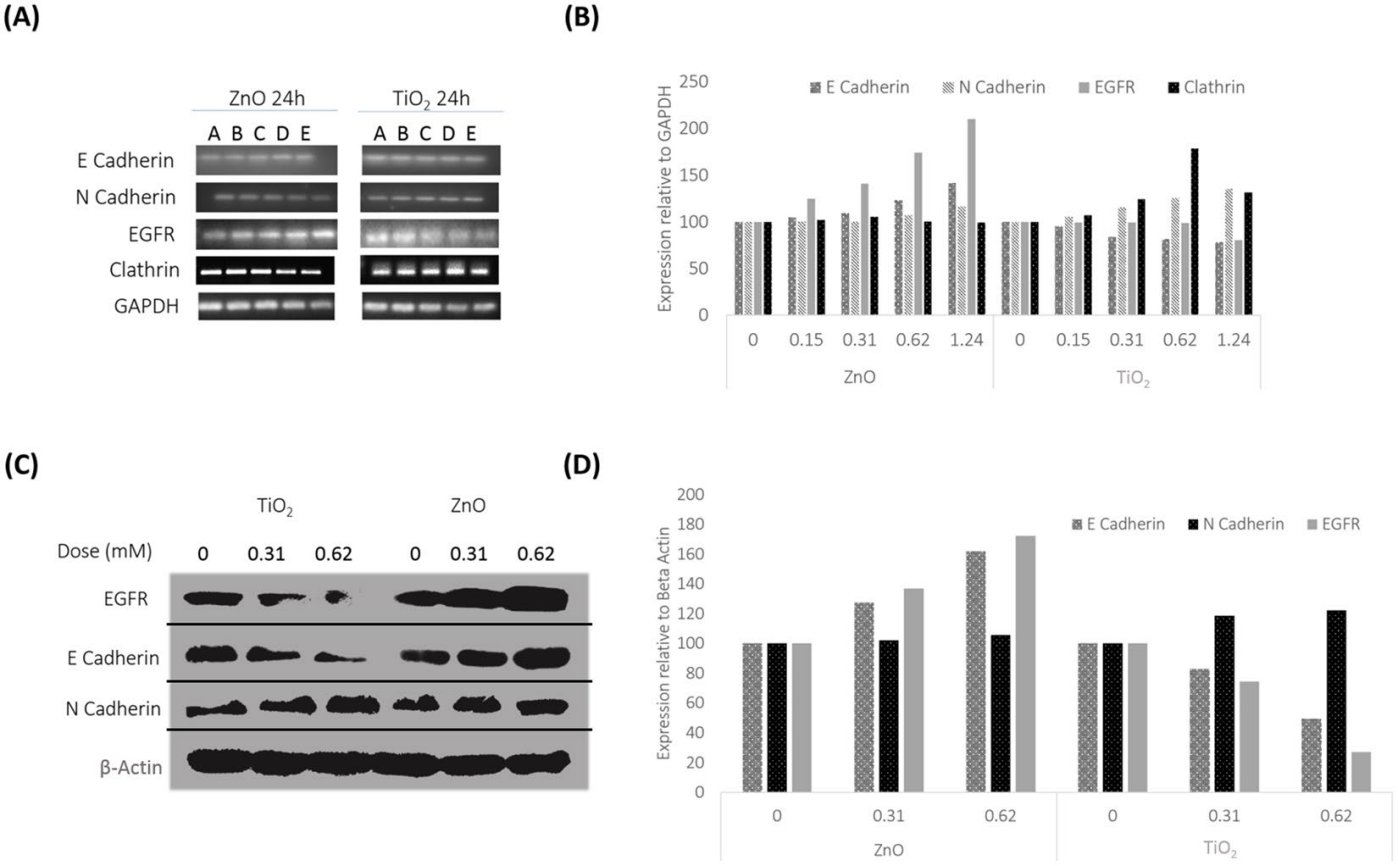
(**A**) Evaluation of EMT by mRNA level expression of E Cadherin, N Cadherin, EGFR and Clathrin. 24 Hours expression pattern of E Cadherin, N Cadherin, EGFR and Clathrin at the mRNA level was documented in a dose dependent manner. Doses; A- 0, B- 0.15, C-0.31, D-0.62 and E-1.24 mM. The best gel of 3 independent experiments is shown here. Results of the independent experiments were following a similar trend in expression. (**B**) Statistical Analysis mRNA level expression of E Cadherin, N Cadherin, EGFR and Clathrin. Dose dependent expression profile is plotted from the best representative gel for E Cadherin, N Cadherin, EGFR and Clathrin, relative to the internal control GAPDH in response to MeOx NP treatment. P values for ZnO treatment are; E Cadherin- 0.000001773 (***), N Cadherin- 2.75E-06 (***), EGFR- 0.000404547 (***) and Clathrin- 9.05E-06 (***). TiO_2_ exposure resulted in P values of; E Cadherin- 0.0001219 (***), N Cadherin- 3.816E-05 (***), EGFR- 4.1E-05 (***) and Clathrin- 0.010 (**). (**C**) Evaluation of EMT through protein level expression of E Cadherin, N Cadherin and EGFR by western blot analysis. Dose dependent expression of E Cadherin, N Cadherin and EGFR is documented to MeOx NP treatment. The best blot of 3 independent experiments is shown here. Results of the independent experiments were following a similar trend in expression. (**D**) Statistical Analysis of EMT markers E Cadherin, N Cadherin along with EGFR at protein level. Dose dependent expression of E Cadherin, N Cadherin and EGFR is plotted from the best representative blot, after normalizing with the internal control; Beta Actin in response to MeOx treatment. P Values for ZnO treatment are; E Cadherin- 0.017406 (*), N Cadherin- 0.00404711 (**) and EGFR- 0.0027940 (**). TiO_2_ exposure resulted in p values of; E Cadherin- 0.037336 (*), N Cadherin- 0.0350571 (*) and EGFR- 0.04914 (*).

E Cadherin upregulated in response to ZnO exposure along with EGFR. EGFR showed a significant upregulation, at 100% expression more than basal level for 1.24 mM of exposure (Fig. 5A). However, in response to TiO_2_, both E Cadherin and EGFR showed a marked downregulation. N Cadherin upregulated in response to both ZnO and TiO_2_, though it was more pronounced in response to TiO_2_. Clathrin, upregulated up till 0.31 mM in response to ZnO NPs, while it stayed elevated till 0.62 mM with TiO_2_ exposure with 75% of control. A further dose treatment at 1.24 mM was documented by a depreciation in expression to 25% more than control level. Densitometric analysis is given in Fig. 5B.

### Protein level expression analysis for EMT

EGFR and E Cadherin downregulated in response to TiO_2_ NPs while they upregulated with ZnO exposure. ZnO treatment rendered 60% increase in E Cadherin expression and 70% increase in EGFR expression at 0.62 mM among all dose points evaluated (Fig. 5C). TiO_2_ exposure resulted in a downregulation of E Cadherin to 40% of control, while EGFR to just 20% of basal expression.

N Cadherin upregulated in response to both ZnO and TiO_2_ exposure, though for the latter, the expression was more pronounced, with about 20% more than the basal expression. Densitometric analysis was represented in Fig. 5D.

### Wound Healing Assay

**Figure 6:**
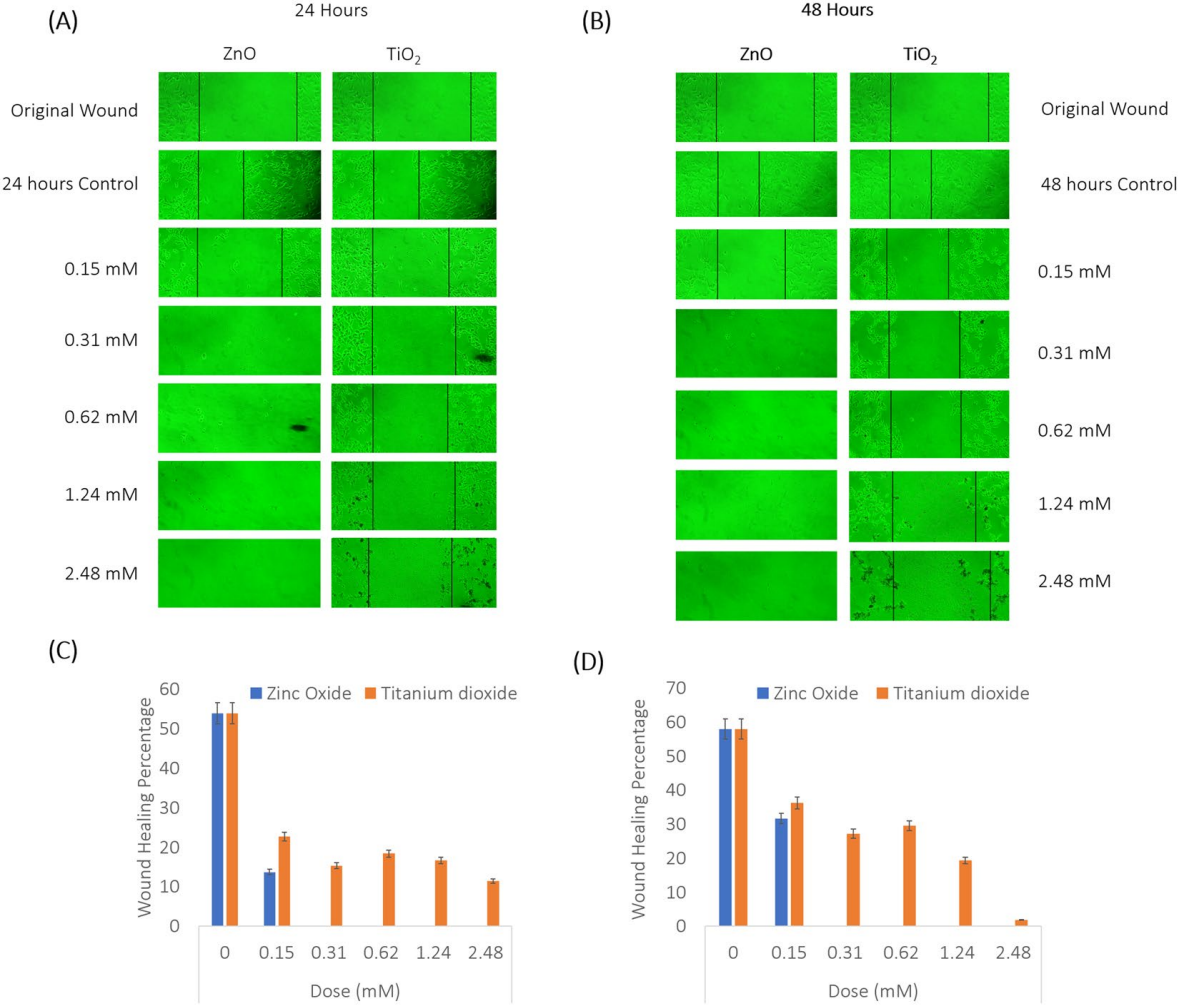
(**A**) Wound healing assay to evaluate Proliferation capacity to ZnO and TiO_2_ NP treatment at 24 hours: Dose dependent documentation by Inverted microscopy is presented. (**B**) Wound Healing Assay recorded for 48 hours. (**C**) Statistical analysis by ImageJ, NIH for 24 hours data. (**D**) Statistical analysis by ImageJ, NIH for 48 hours data. P values calculated are; ZnO 24h- 0.14636 (ns- to be noted; data was not available beyond 0.31 mM in this experimental set up), TiO_2_ 24h- 0.0178037 (*), ZnO 48h- 0.09484 (ns- to be noted; data was not available beyond 0.31 mM in this experimental set up) and TiO_2_ 48h- 0.0013234 (**). The best representative pictures of 3 independent experiments is shown here. Results of the independent experiments were following a similar trend in expression. The average wound healing percentages are indicated. Standard deviation is calculated for 3 different field of view from each independent experiment and denoted by error bars.

The control healing potential of the original wound in an untreated sample, was recorded at 53.89% in 24 hours, while 58% for 48 hours. ZnO exposed cells showed wound healing only at 0.15 mM of exposure, with 13.7% for 24 hours (Fig. 6A) and 31.68% for 48 hours (Fig. 6B). With further increase in dose for ZnO exposure, there was increased cell death with no distinct wound boundary visible. The proliferation capacities in response to TiO_2_ exposure for 24 hours were 22.66%, 15.28%, 18.39%, 16.69% and 11. 46% for doses 0.15, 0.31, 0.62, 1.24 and 2.48 mM respectively. The subsequent proliferation values increased upon incubation at 48 hours up till the dose of 1.24 mM. They were 36.21%, 27.16%, 29.56% and 19.38% for doses 0.15, 0.31, 0.62 and 1.24 mM. At 2.48 mM of dose of TiO_2_ exposed to 48 hours, the proliferation rate dropped to 1.84%. Statistical analysis is provided in Fig. 6C,D.

### Transwell Invasion Assay

**Figure 7:**
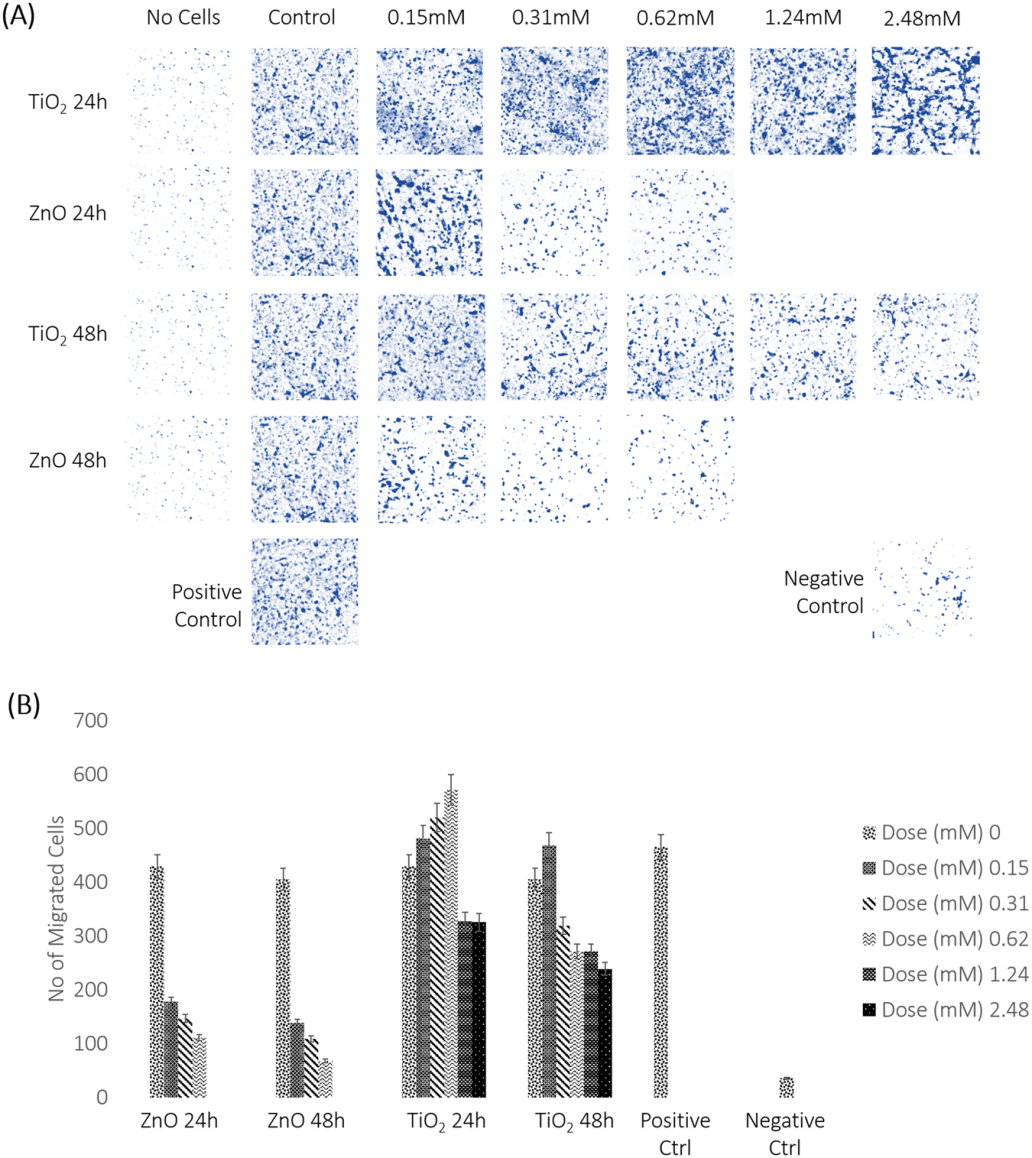
(**A**) Transwell invasion assay to evaluate migration potential in response to MeOx NP treatment. Dose and time dependent documentation of migrated cells against control (untreated membrane) is studied. A positive control and a negative control is also set in the experiment to validate results effectively. The best pictures from 3 independent experiments is shown here. Results of the independent experiments were following a similar trend in expression. (**B**) Statistical analysis of migration potential. Migration capacities are plotted as number of migrated cells in a time and dose dependent manner. Number of migrated cells for the positive and negative control is also depicted. P values are; ZnO 24h- 0.05000 (*), ZnO 48h- 0.0505 (*), TiO_2_ 24h- 0.0004067 (***) and TiO_2_ 48h- 0.00047933 (***). The average number of migrated cells are indicated. Standard deviation is calculated for 3 different field of view from each independent experiment and denoted by error bars.

ZnO exposed cells showed a decline in the number of cells migrating with increase in dose of exposure till 0.62 mM at 24 and 48 hours. Upon further increase in dose, no migrated cells were observed. However, TiO_2_ treated cells, showed an increase in migration with a maximum at 0.62 mM for 24 hours and 0.15 mM for 48 hours (Fig. 7A). The number of migrated cells is plotted against dose of exposure for TiO_2_ and ZnO exposure in Fig. 7B.

## Discussion

We selected two candidate nanoparticles of varying toxicities; ZnO and TiO_2,_ to draw out any differences in cellular responses of A549 cells to their exposure. A similar total viability response was observed of decrease in viability with increase of exposing dose. However, as expected ZnO was more lethal than TiO_2._ With increased incubation periods, viability improves in case of both TiO_2_ and ZnO exposure, especially at 72 hours. This might be because cells in question are alveolar type II cells (A549) which are known to display higher tolerance to stress^36^.

Resazurin reduction assay was performed to gauge the metabolic activity and mitochondrial health of cells exposed to metal oxide nanoparticles. ZnO did confer higher lethality over TiO_2_, with LD_50_ value of 2.26 mM as against 44.15 mM for the latter. Resazurin reduction values were significantly higher than total viability values, for respective doses evaluated, suggesting mitochondrial dysfunction may not be the only cause of death.

Morphological documentation reveals less budding typical of apoptosis and more cells with ruptured plasma membrane with increase in exposure, especially for ZnO treatment. This allied with analysis from trypan blue dye exclusion test and resazurin reduction assay suggest a majorly necrotic mode of death.

We had explored the phosphorylation status of eIF2α to understand if this well-studied unfolded protein stress response is an occurrence in nanoparticle mediated toxicity. A continued phosphorylation of eIF2α, particularly at 48 hours in response to TiO_2_ as compared to ZnO nanoparticles was observed. This is the first report of its kind, discovered by us. Western blot analysis was undertaken and phosphorylation status of eIF2α was investigated with respect to total eIF2α as the control. This has been double checked with another internal control βActin (data not shown in the paper). The Integrated stress response (ISR) merges with the unfolded protein response (UPR) cascade at the PERK sensor (Protein kinase R like endoplasmic reticulum kinase). Sensors within the unfolded protein response cascade are present along the membrane endoplasmic reticulum (ER) membrane. Phosphorylation of eIF2α could thus be an adaptive response by providing the cell with an opportunity to limit deleterious effects of noxious agents and help conserve resources. Expression of specific repair agents such as Hsp70 during this period, can be regulated to aid stress recovery^37^. Over expression of Hsp70, is also known to reverse misfolded proteins including cytoplasmic aggregations such as the stress granules (SG)^38^.

Our analysis of Hsp70 expression at the protein level, revealed, for TiO_2_ exposure, Hsp70 stayed elevated for 24 and 48 hours up till the evaluated dose point of 0.62 mM. ZnO treatment saw an upregulation only up to 24 hours till 0.15 mM. Thereafter with increased incubation to 48 hours Hsp70 expression downregulated. The longer duration of phosphorylation of eIF2α observed with TiO_2_ exposure along with an increase in Hsp70 expression is most crucial. This may provide the cell with systems to counter cellular damages such of misfolded proteins, ultimately increasing the potential for repair in TiO_2_ treated cells.

The phosphorylation status of eIF2α is also a translational regulation event^39^, which may account for the differences in expression profiles of small GTPases between the mRNA and the protein level.

mRNA level expression of small GTPase for both ZnO^35^ and TiO_2_ exposure follow a similar pattern; upregulation of RhoA and Rac1 while a spiked expression for cdc42. The pattern varies however between ZnO and TiO_2_ exposure at the protein level. With cdc42 upregulating upto 60% more than control at 0.62 mM in case of TiO_2_ and downregulating to 40% of control expression at the same dose for ZnO. This can directly be co-related to the increased filopodial phenotypic expression to TiO_2_ not observed with ZnO exposure.

We further, discovered E Cadherin downregulation along with N Cadherin upregulation in response to TiO_2_ exposure, a hallmark for EMT^40^. This is a novel finding. This is not evident with ZnO NP treatment. This is supplemented with the allied EGFR expression that follows suit with E Cadherin^41^. Clathrin is crucial to EGFR internalization^42^. At mRNA level, Clathrin expression does stay elevated up till a dose of 0.62 mM at 78.34% more than control in TiO_2_ exposure. Whereas, clathrin peaks at 0.31 mM with just 5% more than control to ZnO treatment. Thereafter, with further dose exposure, clathrin expression downregulates. These results suggest, with MeOx exposure on A549 cells, clathrin upregulation and its mediated internalization of EGFR, result in degradation of EGFR.

Increased proliferative and migrative capacity of TiO_2_ NP treated cells over ZnO was also recorded through wound healing and transwell invasion assays. Wound healing assay shows a marked preservation in proliferation capacity with TiO_2_ treatment as compared to ZnO. These cells may thus, migrate away from the zone of stress, enabling them to tolerate higher TiO_2_ exposure as compared to ZnO. In toxicity assessments particularly, that of the *in vitro* set up, a zone of stress represents the layers of variant shear stress in a culture vessel^43^. We postulate that adherent cultures exhibit high stress on cells close to the basal membrane, with marked nutrient deprivation and increased steric hindrance through crowding of the cells. The density dependent depletion in cell growth and proliferation is widely documented for confluent cultures^44^. It is possible that epithelial to mesenchymal transition allows cells to float away from the lamina into a region more conducive for survival. Moreover, the regulated release of cytokines and chemokines by stressed cells also less affect the cells away from the high zone of stress at the basal lamina^43^.

Though for all doses evaluated for both ZnO and TiO_2_ exposure, mitotic capacities^45^ lagged with the untreated control, suggesting, MeOx NP treatment does negatively affect cellular proliferation, irrespective of lethality.

Further epithelial to mesenchymal transition, is also known to phosphorylate eIF2α through Protein kinase RNA-like ER kinase (PERK) activating the unfolded protein response (UPR) in response to endoplasmic stress^46^. We have substantially proved that TiO_2_ NP treated cells do have an increased duration for which eIF2α remains phosphorylated as compared to ZnO exposure. This may well be related to the onset of epithelial to mesenchymal transition observed in TiO_2_ NP treated cells, not observed with ZnO exposure.

**Figure 8:**
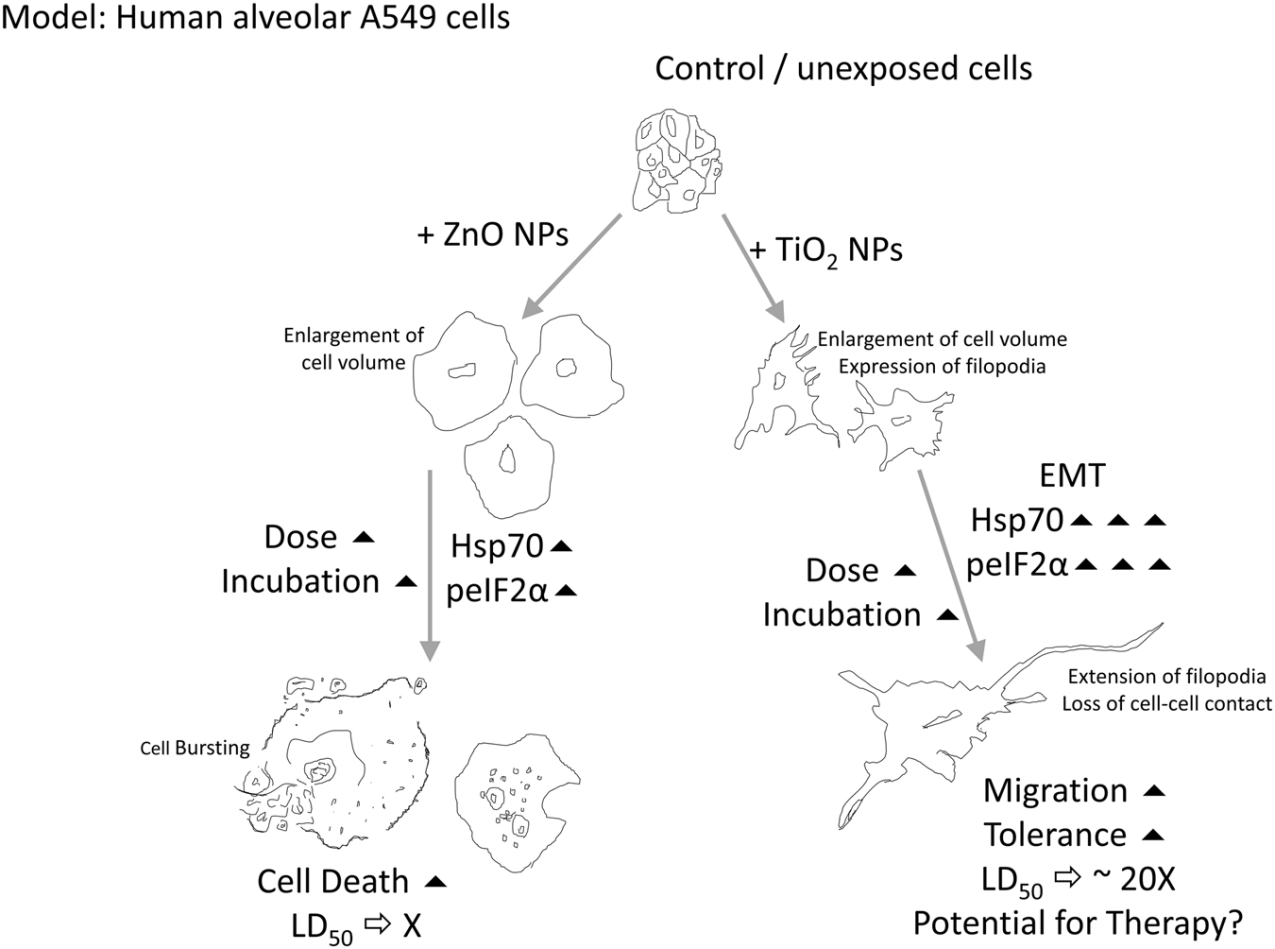
Scope for therapy to nanotoxicity. Extended duration of eIF2α phosphorylation, epithelial to mesenchymal transition and enhanced expression of Hsp70 enable greater tolerance to nanoparticle treatment.

Our data sufficiently proves, A549 cells can withstand a greater dose of exposure from TiO_2_ NPs as compared to ZnO (Fig. 8). LD_50_ value for TiO_2_ nanoparticle treatment on A549 cells is almost 20 times more than that with ZnO. This is due to the epithelial to mesenchymal transition that occurs at the molecular level along with cdc42 expression that renders filopodial extensions. Allied with increased Hsp70 expression and phosphorylation of eIF2α, cellular responses to TiO_2_ NP exposure can open novel routes of therapeutic strategies to alleviate nanoparticle induced stress.

There are already drugs being tested to increase chaperone expression such as Hsp70^47^. The capability of this protein to stabilize denatured protein complexes has developed as an attractive drug development concept. Members of this chaperone family are some of the most ubiquitous and conserved proteins making modelling for response accuracy more convenient along a wide range of systems. Recent research has already uncovered several potential drug molecules that could improve the expression of Hsp70. Derivatives of shikonin and echinochrome can upregulate Hsp70 and reduce cell mortality in response to heat stress, hydrogen peroxide and staurosporine treatment^48^. Exercise has also been implicated in increased Hsp70 expression^49^. Resvaratol, a common ingredient in wine, has proved to increase survivability in mice by upregulating Hsp70^50^. Foods rich in antioxidants such as blueberry and curcumin have also shown a signification upregulation of Hsp70 leading to increased cell recovery and survival^51,52^.

On the other hand, phosphorylation of eIf2α as a target of drug development route has been less studied. Salubrinal, an inhibitor of GADD34 has been recently shown to upregulate phosphorylation of eIF2α^53^. Although its use for any toxicity related therapy has not been investigated. Epithelial to mesenchymal transition has long gained a spotlight for cancer progression^54^. Although this signalling cascade has not been evaluated as a stress revival strategy in normal cells.

Our research discovers three novel routes, that are related and can together confer 20 times of dose tolerance to nanoparticle exposure. Cellular mechanisms such as upregulation of Hsp70, increased phosphorylation of eIf2α and induction of epithelial to mesenchymal transition, can enable human alveolar type II cells to migrate away from a zone of stress in the alveolar lining. They can tolerate a higher level of toxic treatment. These strategies either by themselves or in combination with other novel approaches have huge potential to be developed into therapeutic regimens for nanotoxicity especially in the case of pulmonary distress. A world of raising pollution and aerosolized nano material need the discovery and development of such approaches to combat lethal toxicities to human health.

## Methods

All the general laboratory chemicals were purchased from Sigma-Aldrich (USA) and Himedia (India), antibodies from Cell Signalling (USA), Biolegend (USA) and Abcam (USA). Human broncho-alveolar carcinoma-derived (A549) cells were obtained from National Centre for Cell Sciences, Pune. Characterized nanoparticles were purchased from Sigma, Aldrich (USA). Culture plastic ware were procured from Corning Life Science. All experiments were carried out in triplicates.

### Cell Culture

A549 cells were cultured *in vitro* and maintained in DMEM supplemented with 10% FBS (37 °C with 5% CO_2_). Experiments were conducted at 80% confluence unless otherwise mentioned.

### Charging of Nanoparticles

ZnO and TiO_2_ NPs were dispensed in a stock concentration of 1 mg/ml (12.2 mM ZnO and 12.5 mM TiO_2_). The doses of charge chosen were; control, 12.5 µg/ml (0.15 mM), 25 µg/ml (0.31 mM), 50 µg/ml (0.62 mM), 100 µg/ml (1.24 mM) and 200 µg/ml (2.48 mM) for ZnO and TiO_2_ NPs. Details are given in Table 1. Doses were chosen based on our viability assays^35^ along a range commonly observed at industrial sites^2^. Stocks were prepared in DMEM aseptically and charged for experiments in the chosen doses after vortexing thoroughly to avoid aggregation and to ensure exposure to nano-scale particles. Serum was added separately to ensure a consistent 10% concentration throughout various experiments.

**Table 1:** Design of Resazurin reduction assay for testing nanoparticle mediated mitochondrial dysfunction.

Dose (mM)	NP vol (µl)	Stock (mM)	DMEM (µl)	10% serum (µl)	Cells seeded
0 (Ctrl+)	0	0	1350	150	Yes
0.15	22.5	10	1327.5	150	Yes
0.31	46.5	10	1303.5	150	Yes
0.62	93	10	1257	150	Yes
1.24	186	10	1164	150	Yes
2.48	372	10	978	150	Yes
4.96	744	10	606	150	Yes
9.92	744	20	606	150	Yes
19.84	974	30	376	150	Yes
39.68	584.4	100	765.6	150	Yes
79.36	1168.8	100	181.2	150	Yes
Neg (−)	0	0	1350	150	No

This method of charging nanoparticles ensures suspension in the nano scale at the time of exposure to cells.

### Cell Viability Assay

A549 cells were exposed to different concentration of NPs (0.15 mM, 0.31 mM, 0.62 mM, 1.24 mM and 2.48 mM) for 24 h, 48 h and 72 h. Cell growth and proliferation was monitored by determining the cell number using neubauers-hemocytometer following trypan blue dye exclusion test^55^.

### Mitochondrial activity Assay

Cell viability was assayed using Resazurin as per Santimano *et al*.^35^. Cells were seeded at a low seeding density of 2.5 × 10^4^ cells per well in a 24 well plate. This was done to ensure resolution along the range of doses tested. Post 24 hours of seeding, fresh media was added maintaining 10% serum to better dispense nanoparticles away from aggregation. Nanoparticles were added after thorough vortex from the least concentrated stock to maintain a minimum aggregation at charging, this method was developed by us for proper dispersion of NPs at the time of charging to mimic the dispersion typical of aerosolized NPs. The design is detailed in Table 1. Resazurin was added in a working concentration of 440 µM. After 4 hours of incubation at 37 °C, positive difference (absolute value) in absorbance at wavelength of 580 nm and 615 nm of each well culture against control (+) was monitored and the percentage resazurin reduction was calculated and reported as a measure of toxicity. The limiting value corresponding to 0% reduction was obtained by measuring OD_580_–OD_615_ of negative control. Percentage Resazurin reduction was used to calculate dose at 50% death (LD_50_).

### Cell Morphology Documentation

Cells were charged with nanoparticles and incubated for time periods of 24 and 48 hours respectively. The monolayer was washed with sterile phosphate buffered saline and fixed with 3.7% formaldehyde for 2 minutes. This was followed by permeabilization with 100% ice cold methanol and staining with Hoechst (33342, thermos fischer) for 15 minutes. Fluorescent pictures of nuclei were captured by an inverted microscope to aid visualization of nucleus. A DAPI filer was used that allowed for illumination of light around 340–380 nm and emission around 465 nm.

### Studying mRNA level expression by RT PCR

Total RNA was isolated by TRIzol reagent (Invitrogen, Carlsbad, CA, USA), following the manufacturer’s instructions. RNA extracted was reverse transcribed and cDNA synthesized using the Bioline cDNA synthesis kit. cDNA was amplified by PCR. Resolution was done using 1.2% agarose gel electrophoresis (TBE buffer) with ethidium bromide staining, photographed under ultraviolet light (BioRad) and analysed by densitometry. The quantity of each transcript was normalized to that of GAPDH, which served as the internal control.

### Small GTPases

Primers: cdc42- 5′-gcccgtgacctgaaggctgtca-3′ (sense); 5′-tgcttttagtatgatgccgacacca-3′ (anti-sense), Rac1- 5′-ggagaatatatccctactgtc-3′ (sense); 5′-cttcttctccttcagtttct-3′ (anti-sense), RhoA- 5′-cccagataccgatgttatac-3′(sense); 5′-aacctctctcactccatctt-3′ (anti-sense) and GAPDH- 5′-agaacatcatccctgcctctac-3′ (sense); 5′-ctgttgaagtcagaggagacca-3′ (anti-sense).

### EMT Assessment

Primers: N Cadherin- 5′-gatgtttacagtgcagtctt-3′ (sense); 5′-actgactcctcagttaaggt-3′ (anti-sense), E Cadherin- 5-aggagctgacacaccccctgt-3′ (sense); 5′-catcgtccgcgtctgtggct-3′ (anti-sense), Clathrin- 5′-gaccgggctcatattgctca-3′ (sense); 5′-tctgacggatgttggcagac-3’ (anti-sense), EGFR- 5′-caagtgtaagaagtgcgaagg-3′ (sense); 5′-cagaggaggagtatgtgtgaagg-3′ (anti-sense) and GAPDH- 5′-agaacatcatccctgcctctac-3′ (sense); 5′-ctgttgaagtcagaggagacca-3′ (anti-sense).

### Studying the protein level expression by Western blot analysis

After cell lysate (40 µg) was resolved by 12% SDS-PAGE the western blot was carried out as per Sarkar *et al*.^56^. In brief, the PVDF membranes were washed with tris-buffered saline followed by blocking with 5% non-fat dried milk or 3% BSA as was suited. The membranes were incubated at 4 °C overnight with primary target specific antibodies.The membranes were then incubated with secondary antibodies coupled to horseradish peroxidase for optimised time periods at room temperature. The membranes were washed in combinations of TBS and TBST at room temperature. Immunoreactivities were detected by ECL reagents (Amersham GE Healthcare). Expression of target proteins was normalized to β- Actin.

### Primary antibodies

cdc42 (Cell Signalling- 1:500), Rac1 (Cell Signalling- 1:750), RhoA (Cell Signalling- 1:750), Hsp70 (Sigma Aldrich- 1:4000), eIF2α (Cell Signalling- 1:2000), Phospho-eIF2α (Cell Signalling- 1:1000), EGFR (Cell Signalling- 1:500), E Cadherin (Biolegend- 1:100), N Cadherin (Biolegend- 1:100) and β-Actin (Cell Signalling- 1:2000).

### Transwell Invasion Assay

Transwell chambers were prepared by coating them with 50 µl of 30 µg/ml of matrigel. Commercial matrigel stock was diluted with DMEM as required. Coated chambers were incubated at 37 °C for 24 h. 100 µl of 24 h nanoparticle treated cells, containing 2 × 10^5^ cells/ml ware suspended in DMEM with 2% serum and added onto the upper chamber. 700 µl of DMEM with 20% serum was added onto the lower chamber. Nanoparticle doses evaluated include 0.15, 0.31, 0.62, 1.24 and 2.48-mM of ZnO and TiO_2_ NPs. Upper chamber was placed over the lower chamber and incubated at 37 °C for 8 hours. A positive control is exacted by further reducing the serum concentration in the upper chamber to 0.5%, this increases the concentration difference across the membrane and expedites the rate of migration. A negative control was exacted by resuspending untreated control cells in phosphate buffered saline, this restrains migration owing to lack of nutrition. Post incubation, media from upper chamber is removed and the chamber is washed twice in PBS. Cells were fixed by adding formaldehyde (3.7% in PBS) both in upper and lower chamber for 2 minutes. Formaldehyde was removed, and the chambers were washed twice in PBS. Cells were then permeabilized with 100% ice cold methanol for 20 minutes. Methanol was removed from chambers and they were washed twice with PBS. Staining was done by incubating chambers with 2% fresh crystal violet for 20 minutes. Non-migrated cells were gently scraped using cotton swabs and removed. Migrated cells were photographed and counted.

### Wound Healing Assay

A wound was created in the monolayer by a 200 µl tip. The original wound was photographed. Samples were treated while maintaining a control. Doses evaluated are 0.15, 0.31, 0.62, 1.24 and 2.48-mM respectively for ZnO and TiO_2_ NPs. Post 24 h and 48 h incubation with nanoparticles, the wound was washed with PBS and re-photographed. Gaps in each of the pictures were quantified and percentage proliferation of the treated samples were calculated relative to the control.

## Electronic supplementary material

Supplementary Dataset
