# Supplementary material for: Epithelial to Mesenchymal transition, eIF2α phosphorylation and Hsp70 expression enable greater tolerance in A549 cells to TiO2 over ZnO nanoparticles
Source: Sci Rep. 2019 Jan 24;9:436. doi: 10.1038/s41598-018-36716-2 (PMC6346025; doi:10.1038/s41598-018-36716-2)
Supplement: Supplementary file 1 — Supplementary Dataset [file 41598_2018_36716_MOESM1_ESM.pdf]

Title of Manuscript

Epithelial to Mesenchymal transition, eIF2α phosphorylation and Hsp70 expression enable greater tolerance in A549 cells to TiO_2 over ZnO nanoparticles.

Author Details

Ansie Martin[1|] and Angshuman Sarkar[1*]

Author Affiliation | First Author

* Corresponding Author

Email : asarkar@goa.bits-pilani.ac.in Mobile: +91 9503384704

1. CMBL, Department of Biological Sciences, Birla Institute of Technology and Sciences, K K Birla Goa Campus, Sancoale, South Goa - 403726

Original Files listed below

Figure 2 A: **Dose and time dependent morphological documentation of MeOx NP treatment by Hoechst Staining.**

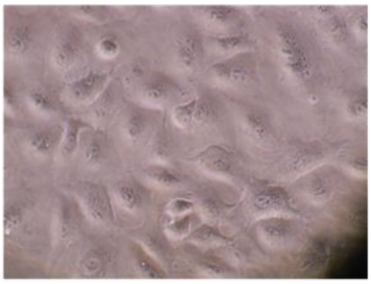
Control 24h
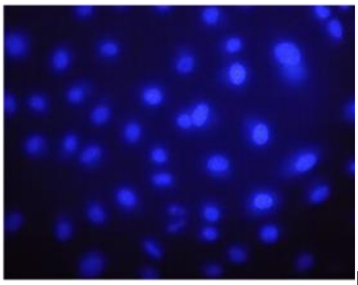
Hoechst 24h

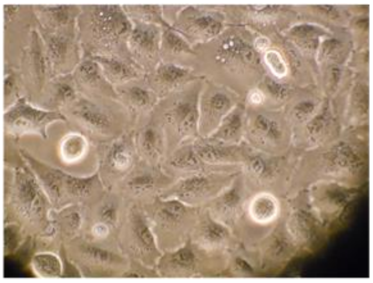
0.15mM ZnO treatment 24h
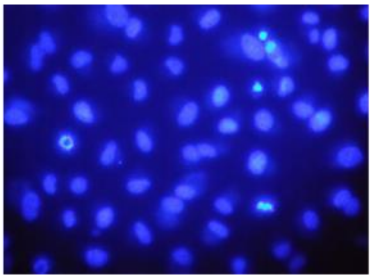

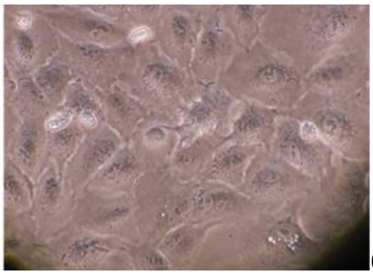
0.31mM ZnO treatment 24h
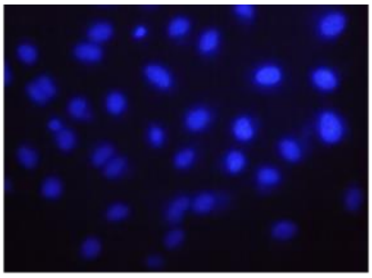

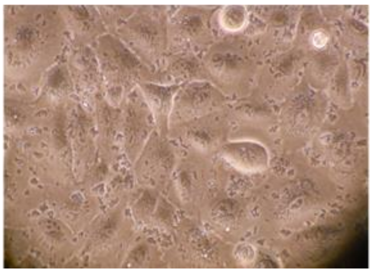
0.62mM ZnO treatment 24h
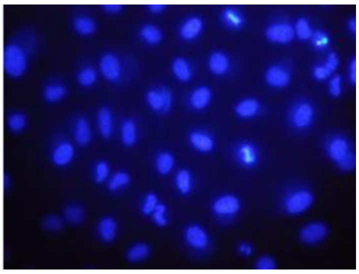

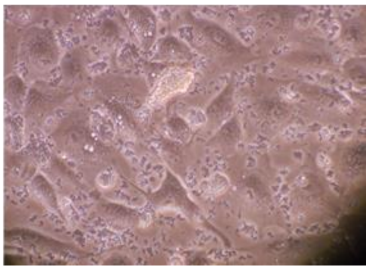
1.24mM ZnO treatment 24h
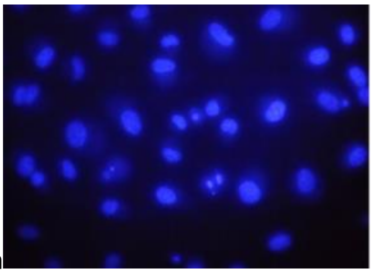

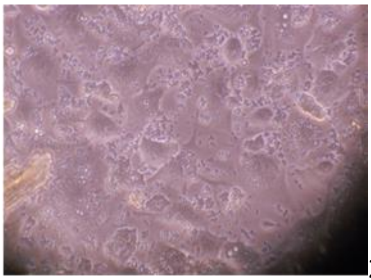

2.54mM ZnO treatment 24h

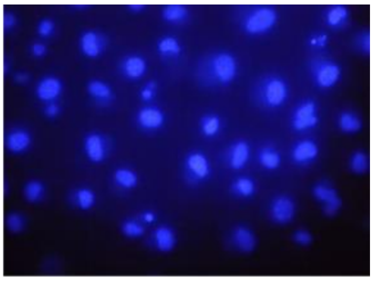

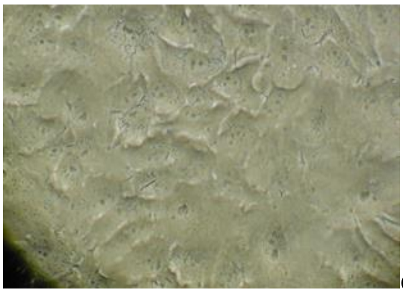

control 48h

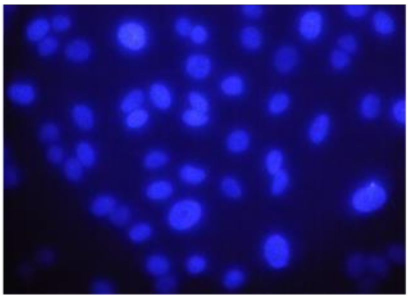

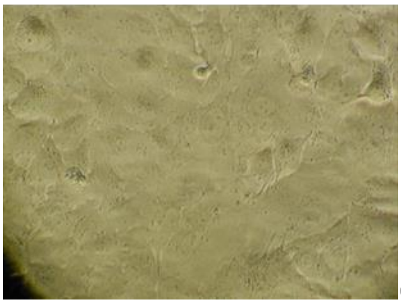

0.15mM ZnO treatment 48h

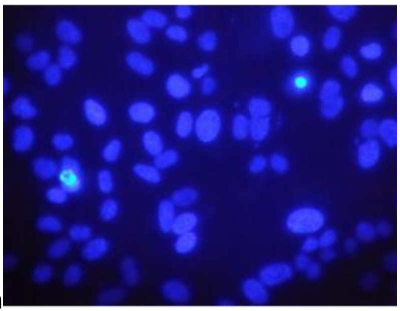

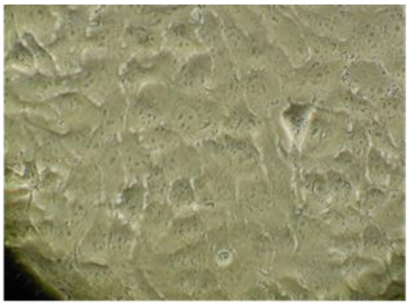

0.31mM ZnO treatment 48h

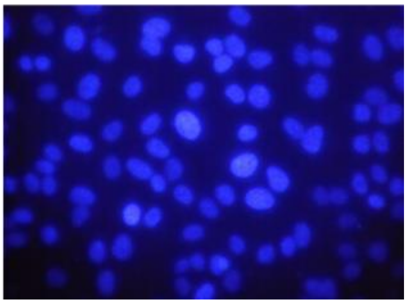

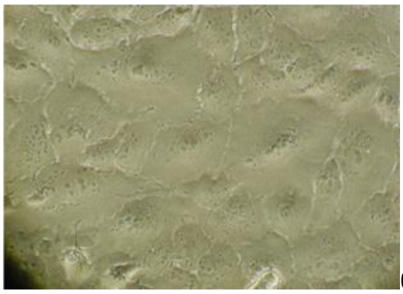

0.62mM ZnO treatment 48h

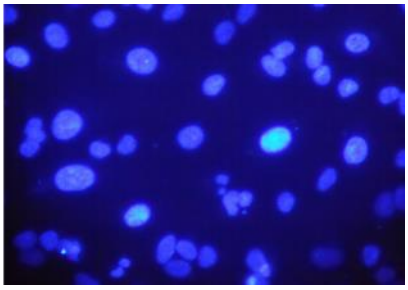

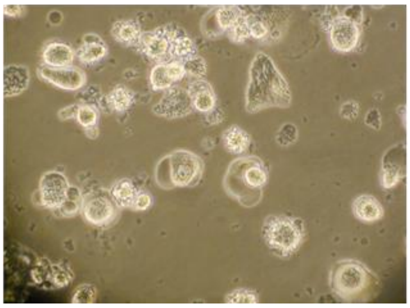

1.24mM ZnO treatment 48h

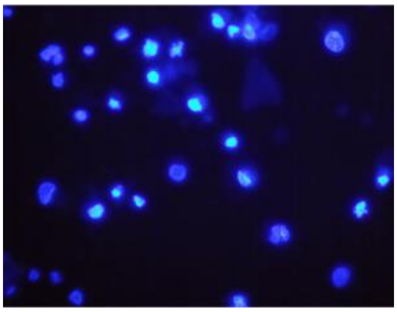

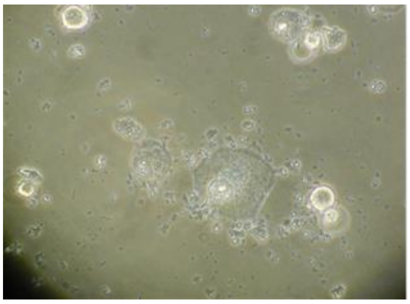

2.54mM ZnO treatment 48h

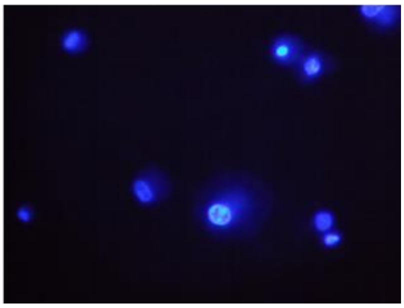

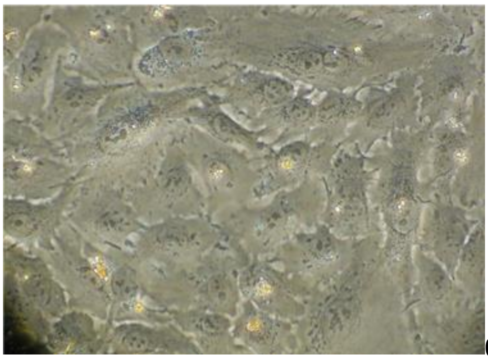

0.15mM 24h TiO_2

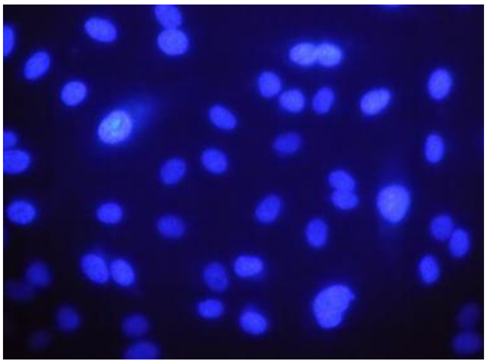

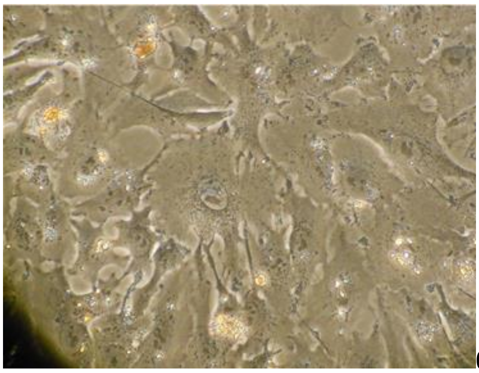

0.31mM 24h TiO_2

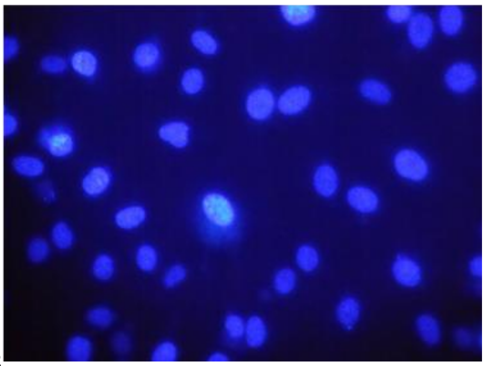

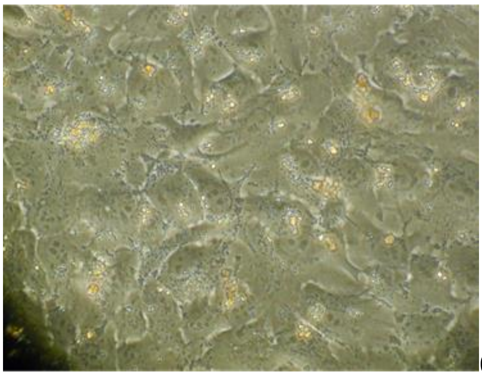
0.62mM 24h TiO_2
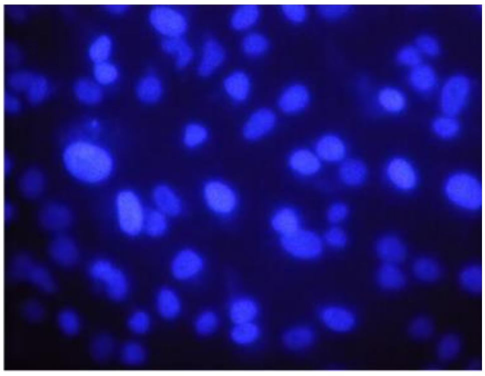

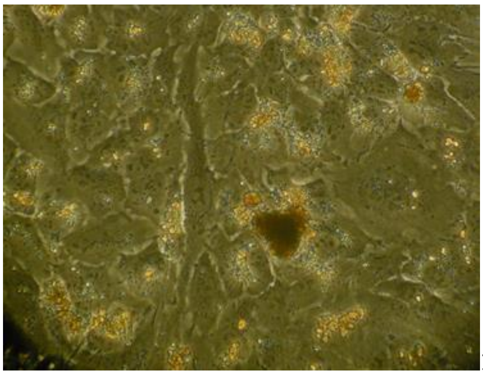
1.24mM 24h TiO_2
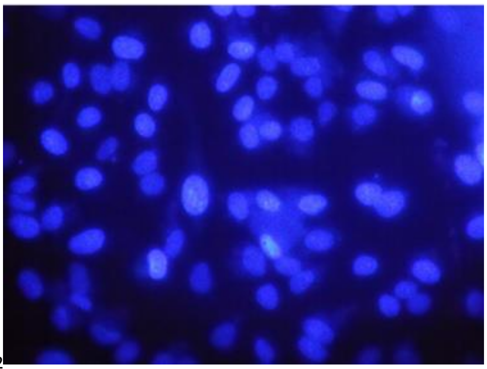

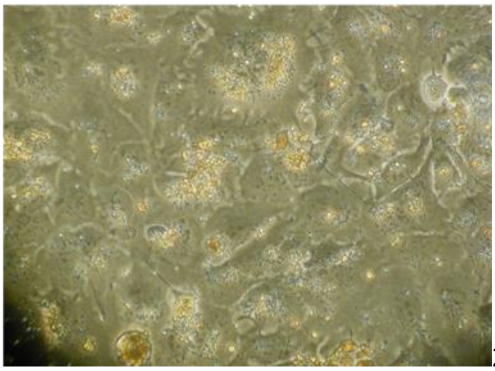
2.54mM 24h TiO_2
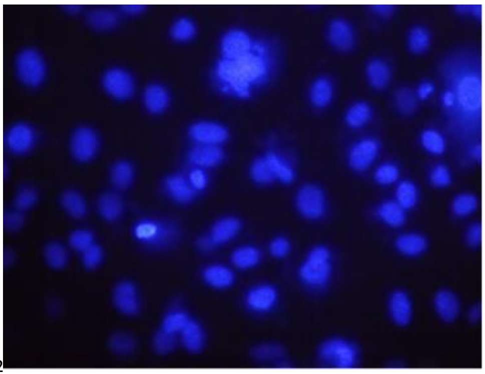

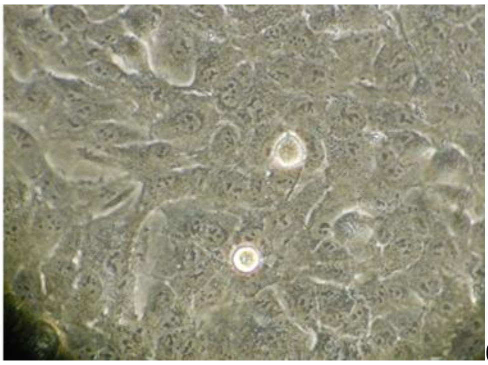
0.15mM 48h TiO_2
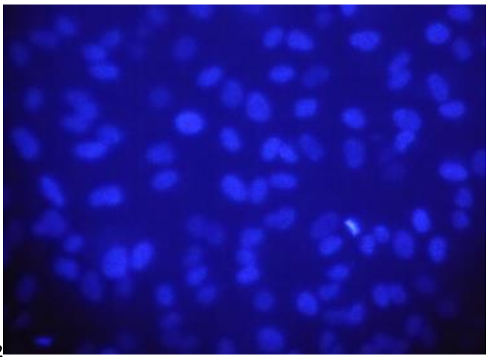

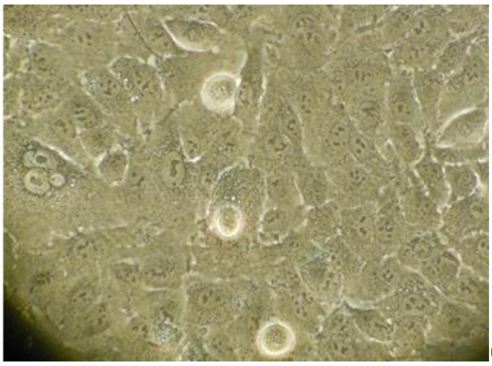

0.31mM 48h TiO_2

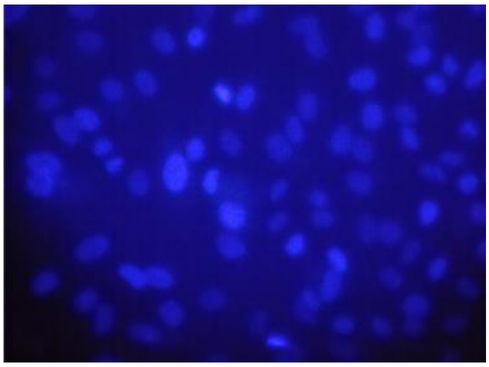

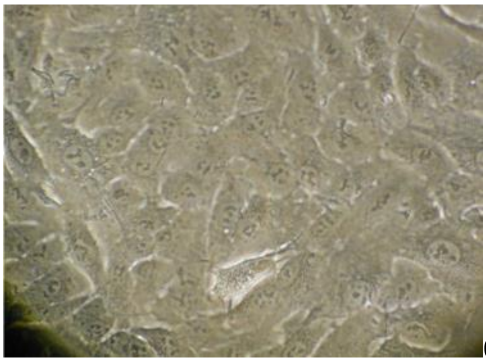

0.62mM 48h TiO_2

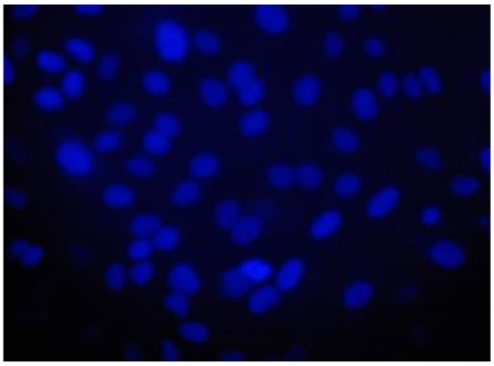

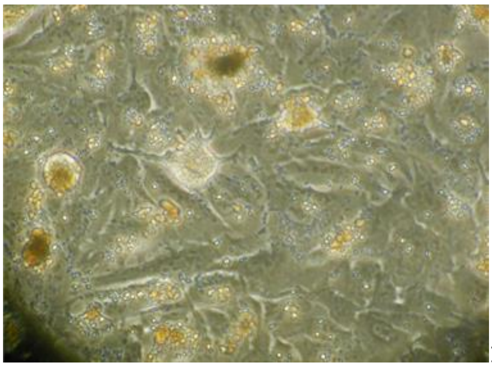

1.24mM 48h TiO_2

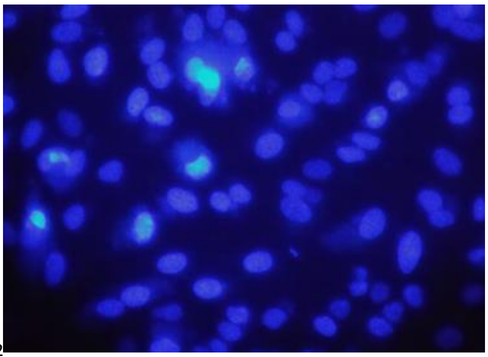

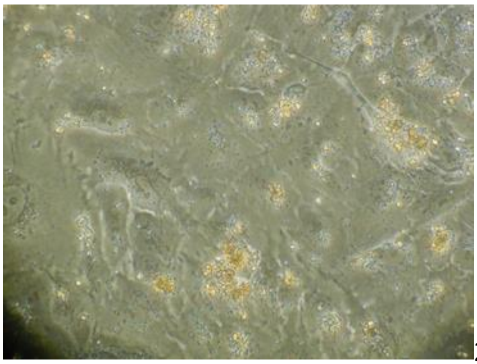

2.54mM 48h TiO_2

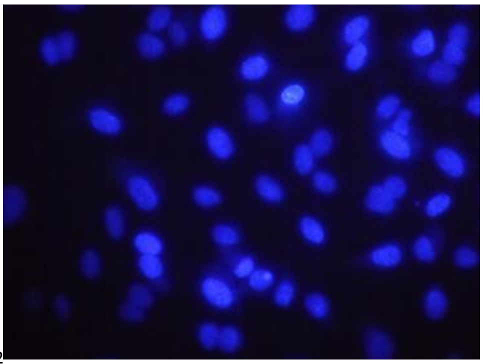

Figure 2 B: **Dose dependent morphological documentation of a single cell to MeOx NP treatment**.

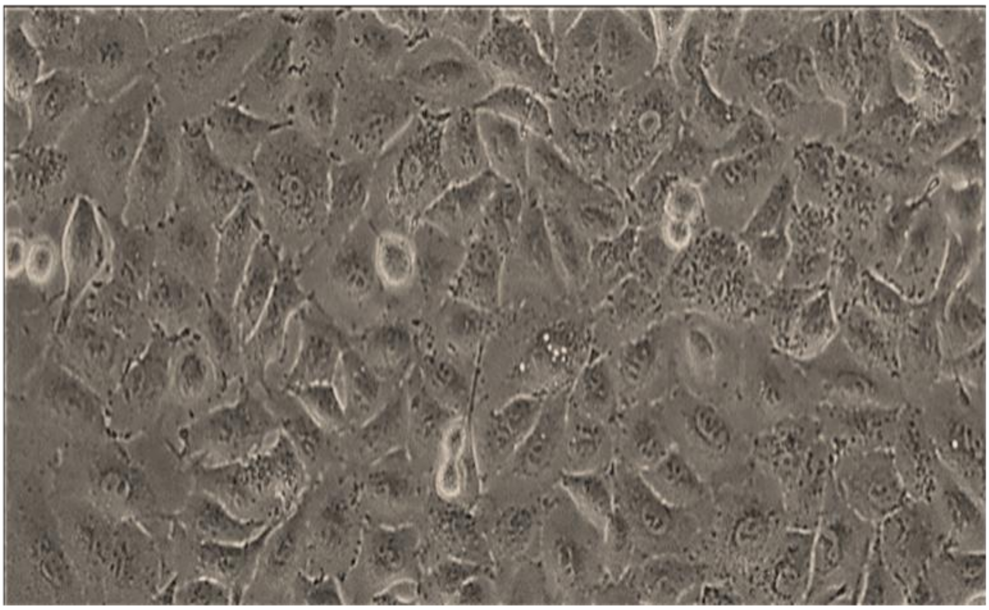

control

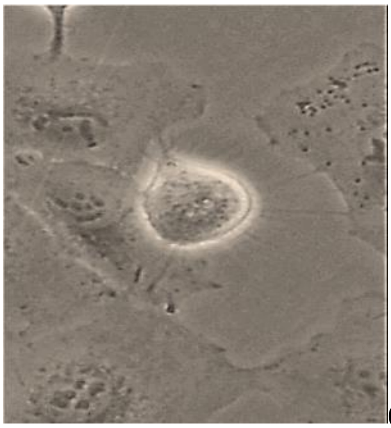

0.15mM ZnO

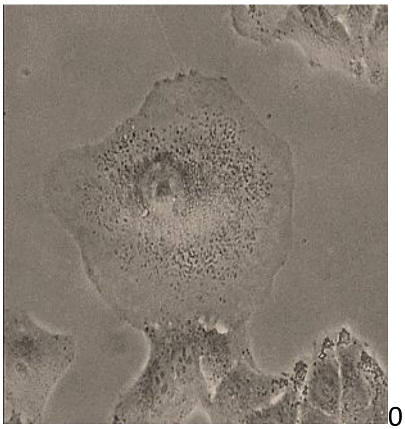

0.31mM ZnO

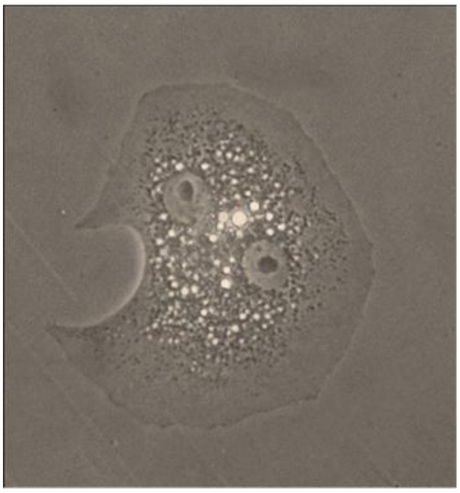

o.62mM ZnO

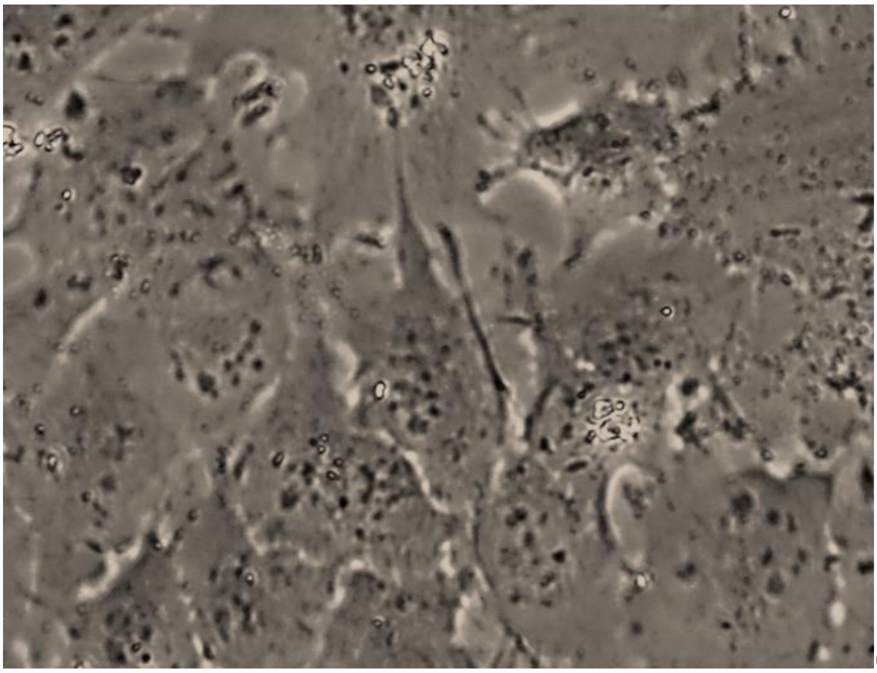
0.15mM TiO_2

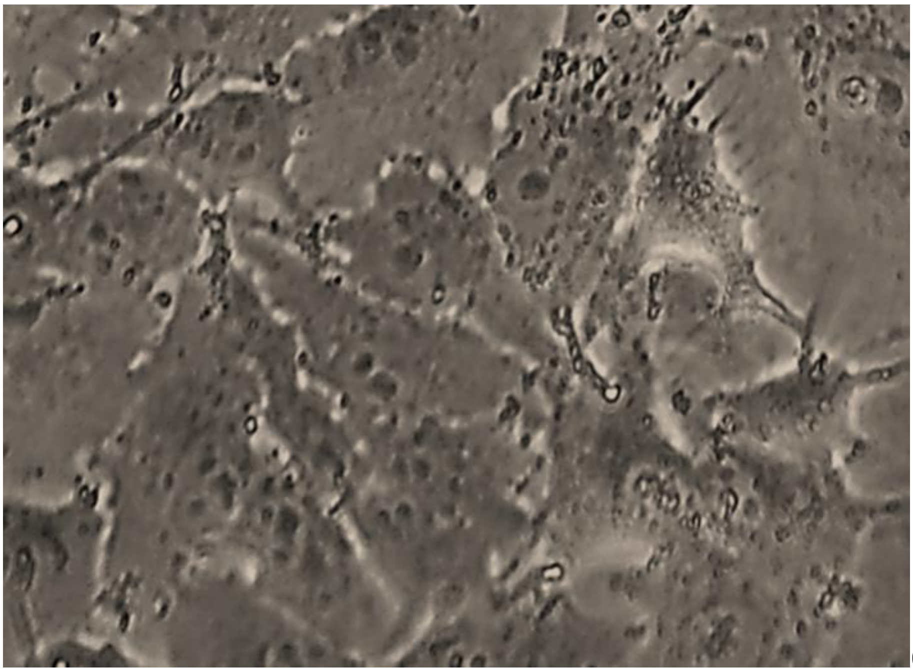
0.31mM TiO_2

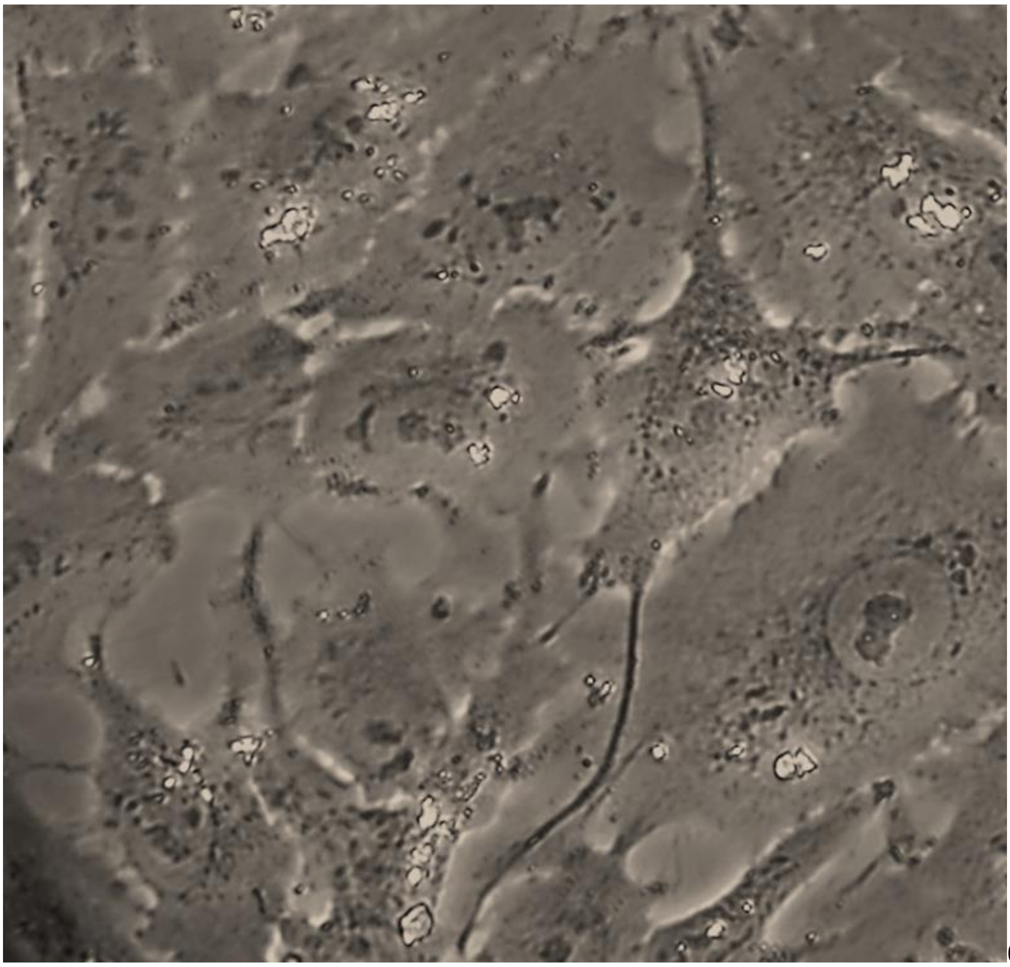
0.62mM TiO_2

Figure 3 A: **Small GTPase expression at mRNA level by RT PCR**.

A = 0.15mM, B = 0.31mM, C = 0.62mM

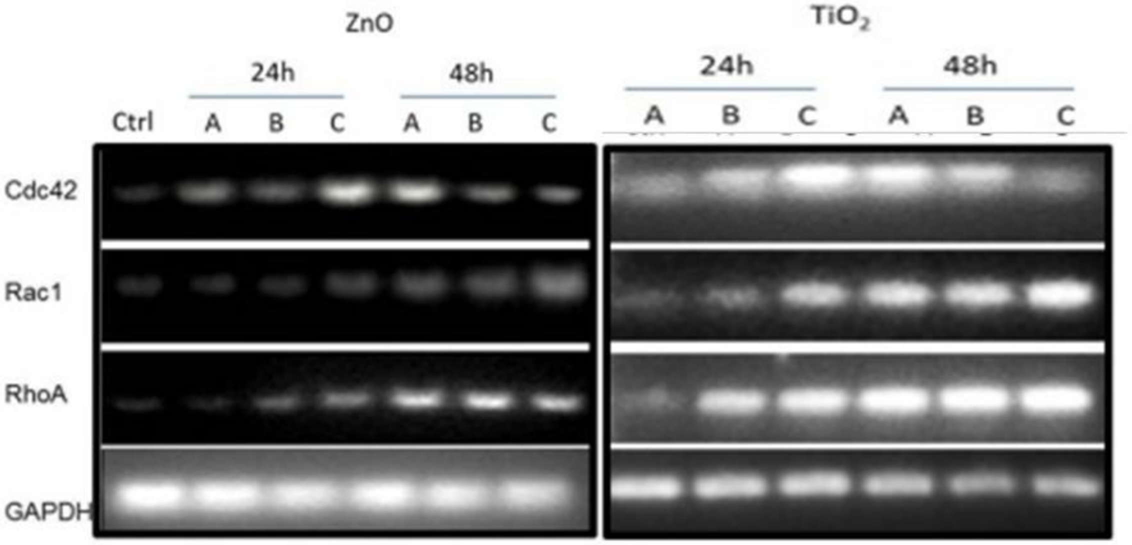

Figure 3 C: **Small GTPase expression at protein level by western blot analysis.**

A = 0.15mM, B = 0.31mM, C = 0.62mM

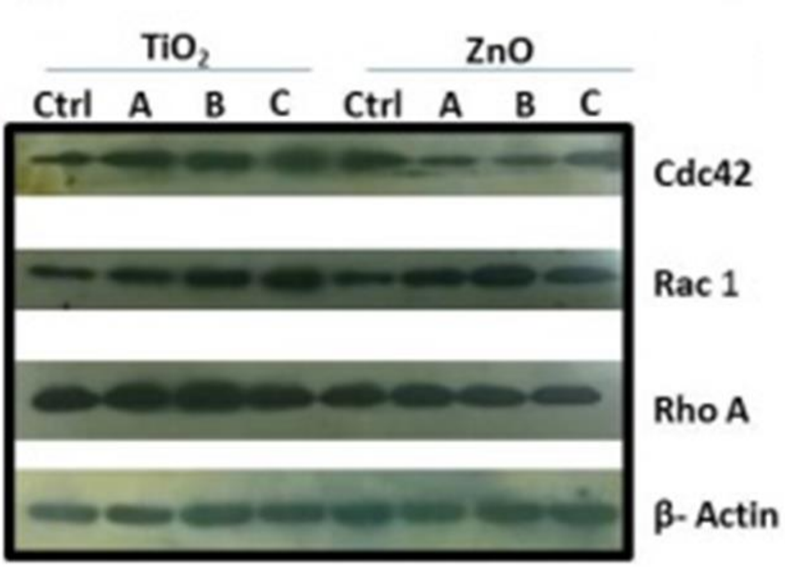

Figure 4 A: **Evaluation of the phosphorylation status of eIF2α by western blot analysis.**

B= 0.31mM, C=0.62mM

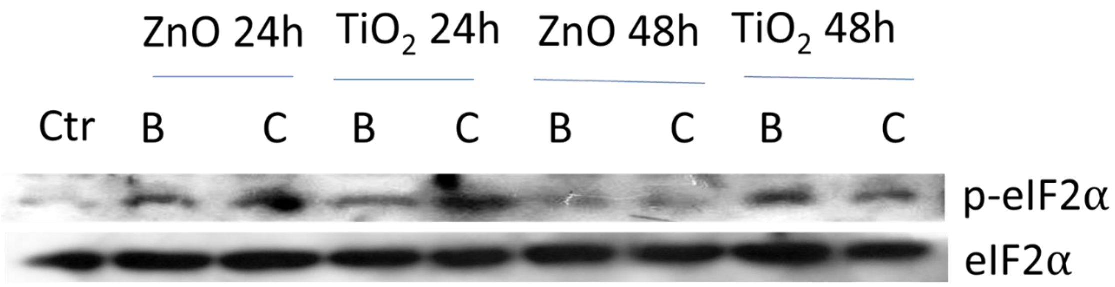

Figure 4 C: **Analysis of the Hsp70 expression at the protein level by western blot analysis.**

A= 0.15mM, B=0.31mM, C=0.62mM

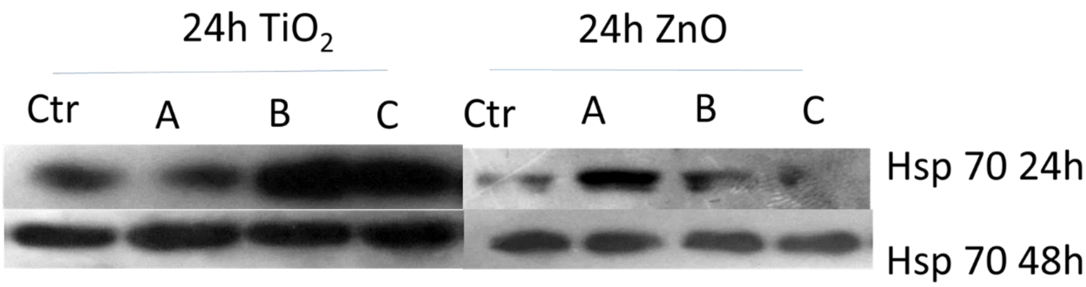

Figure 5 A: **Evaluation of EMT by mRNA level expression of E Cadherin, N Cadherin, EGFR and Clathrin**.

A= 0.15mM, B=0.31mM, C=0.62mM, D=1.24mM, E=2.54mM

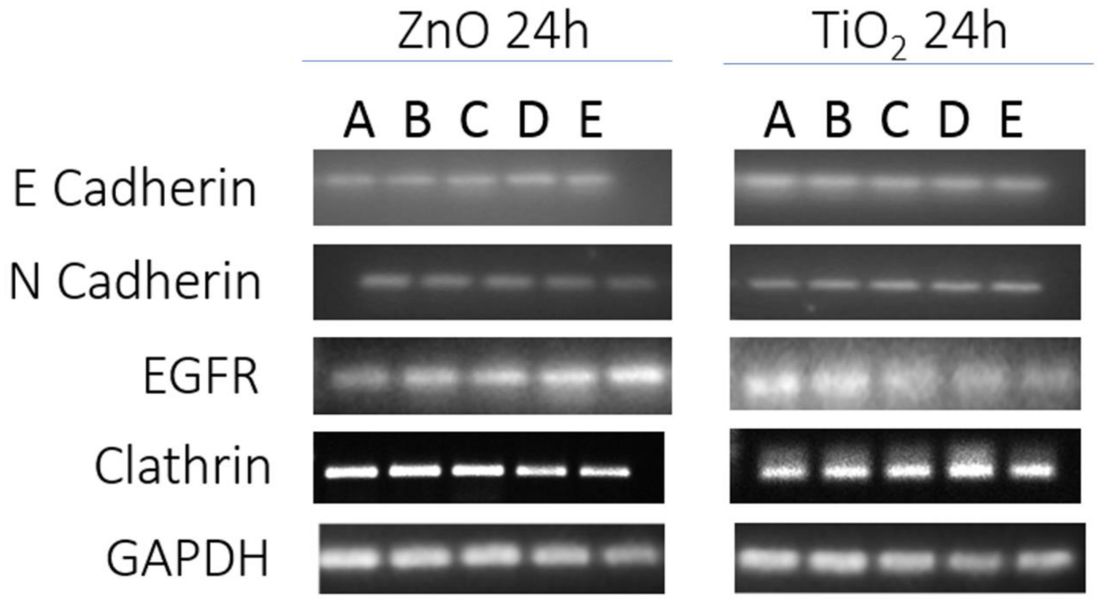

Figure 5 C: **Evaluation of EMT through protein level expression of E Cadherin, N Cadherin and EGFR by western blot analysis.**

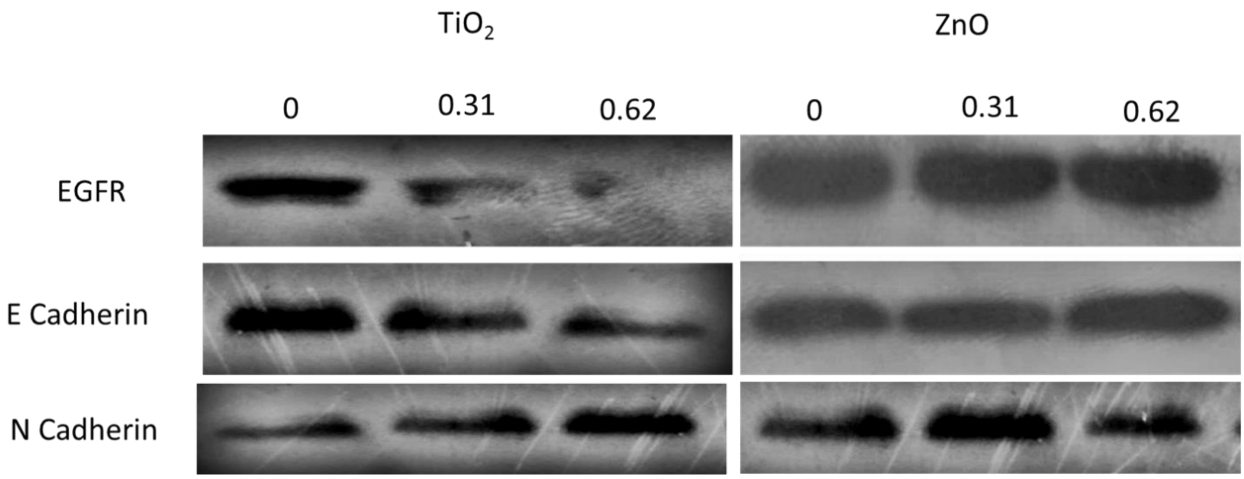

Figure 6: A- **Wound healing assay** to evaluate Proliferation capacity to ZnO and TiO_2 NP treatment at 24 hours: Dose dependent documentation by Inverted microscopy is presented. B- Wound Healing Assay recorded for 48 hours.

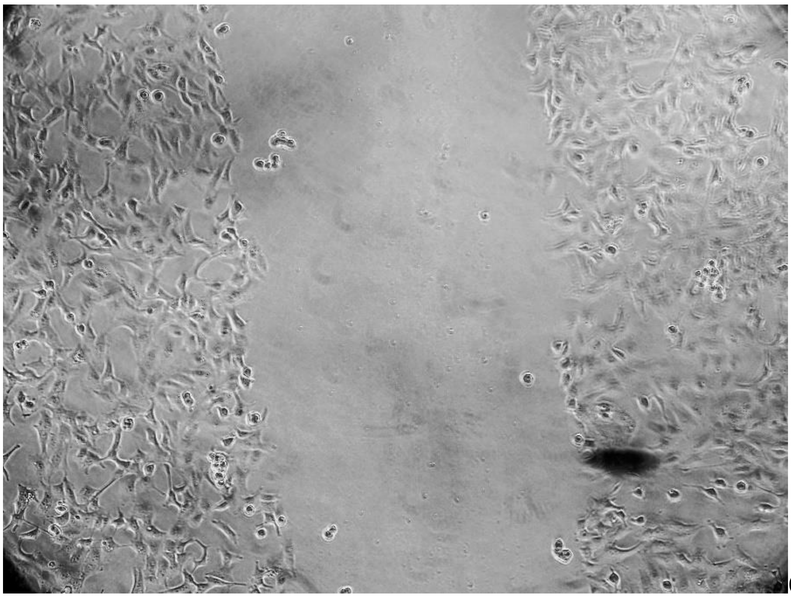
Original wound

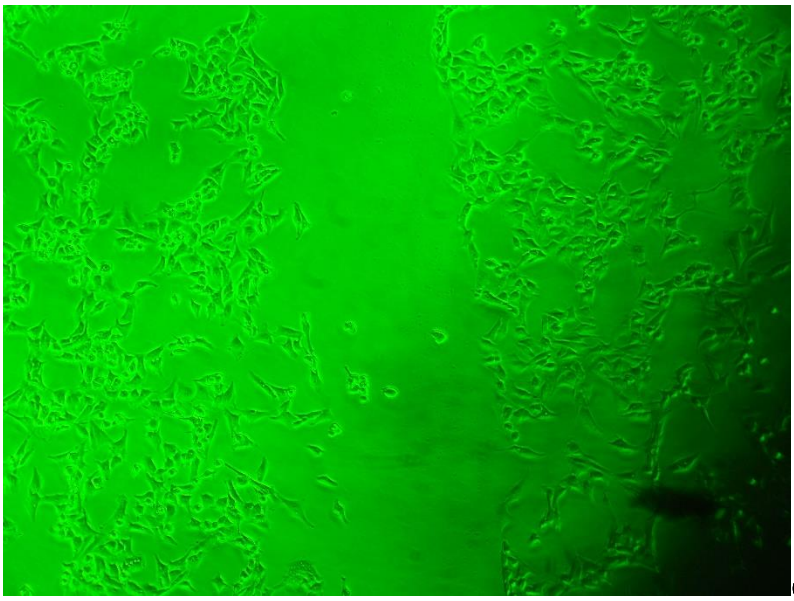
Control 24 hours

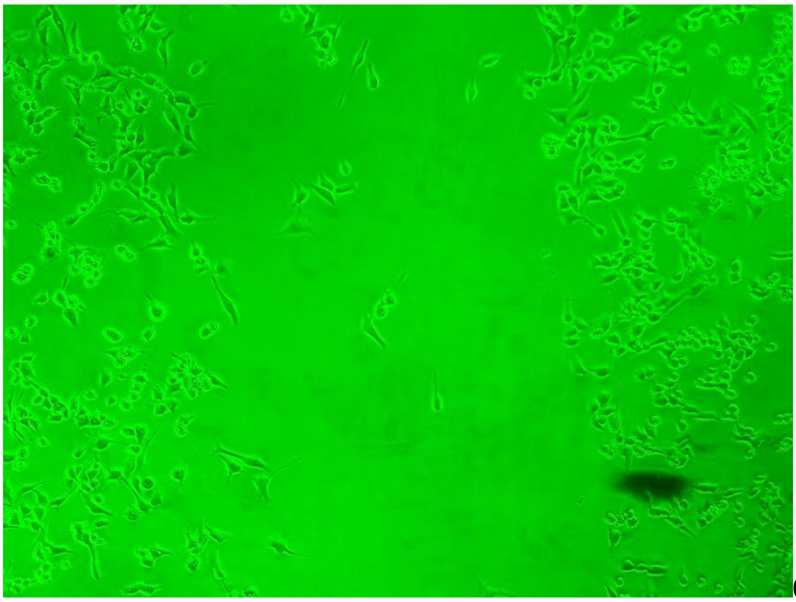
0.15mM ZnO 24h

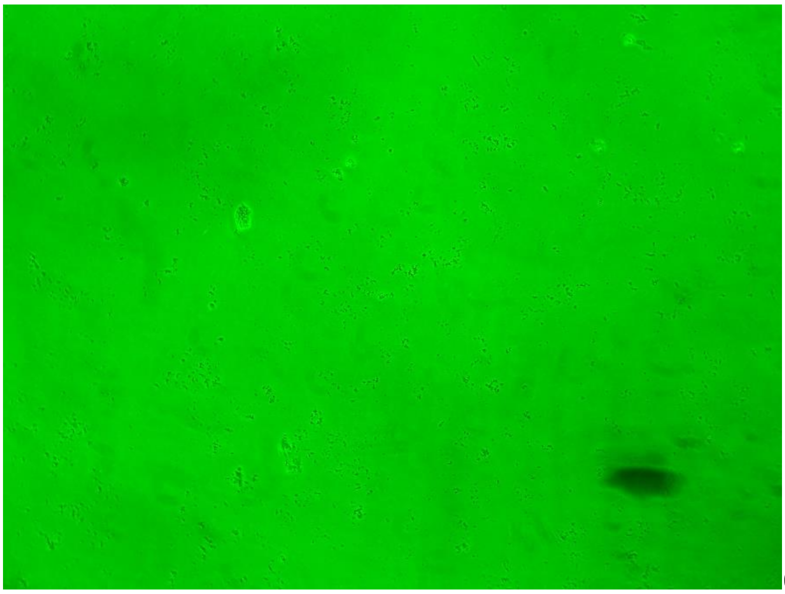
0.31mM ZnO 24h

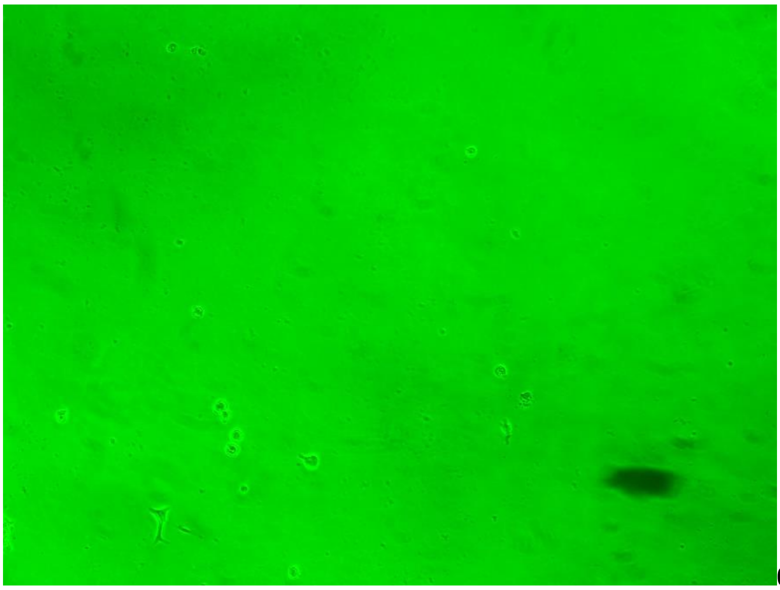
0.62mM ZnO 24h

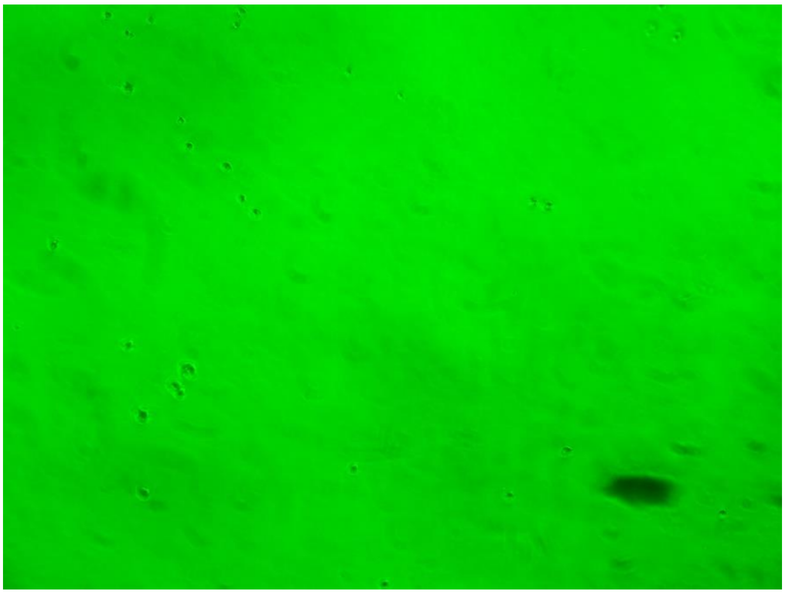
1.24mM ZnO 24h

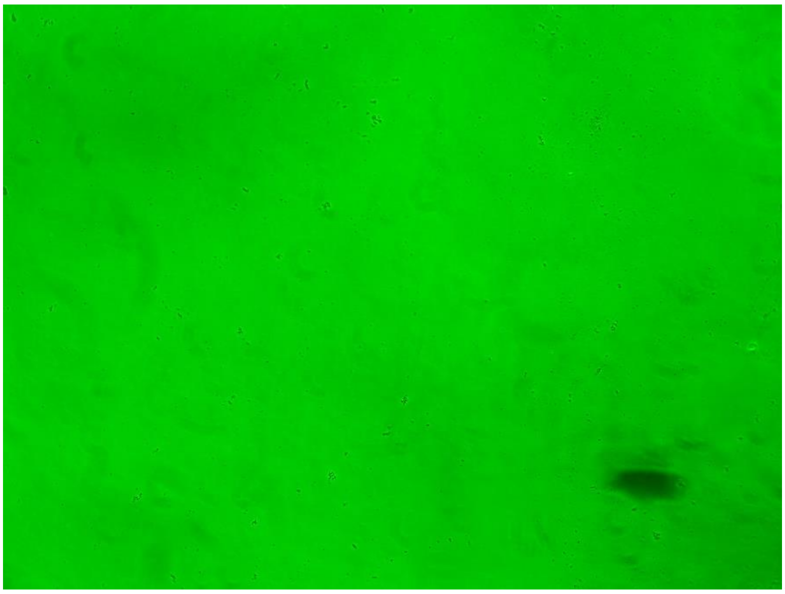
2.54mM ZnO 24h

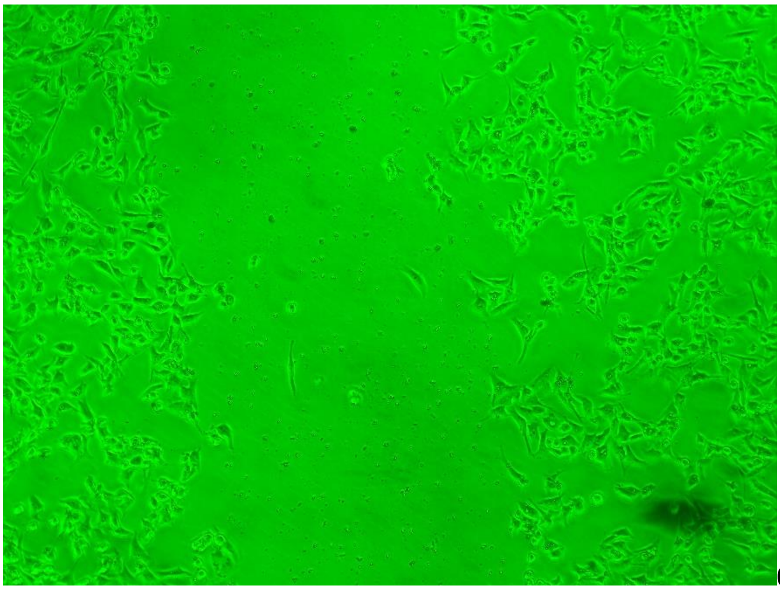
0.15mM TiO_2 24h

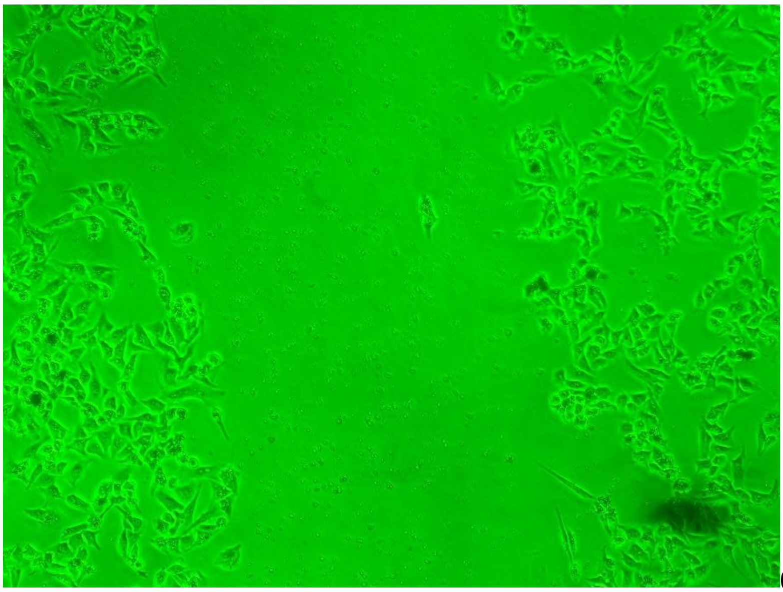
0.31mM TiO_2 24h

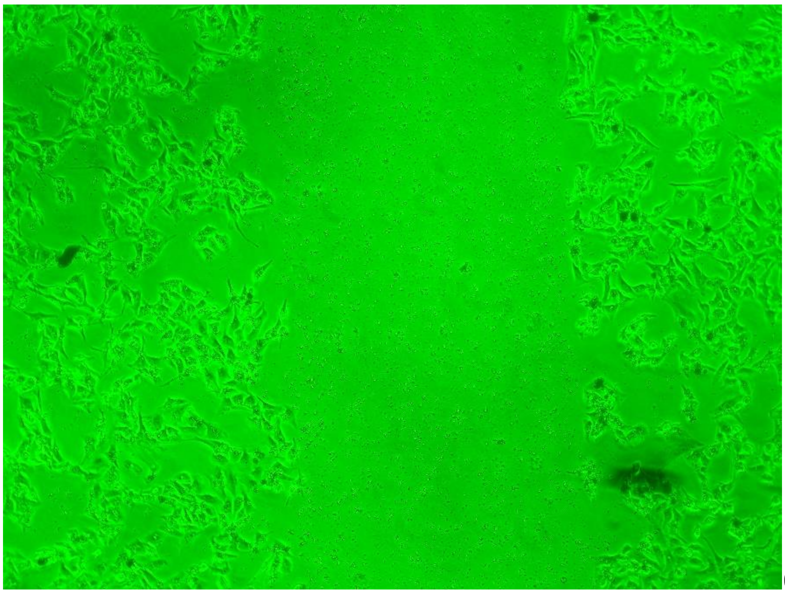
0.62mM TiO_2 24h

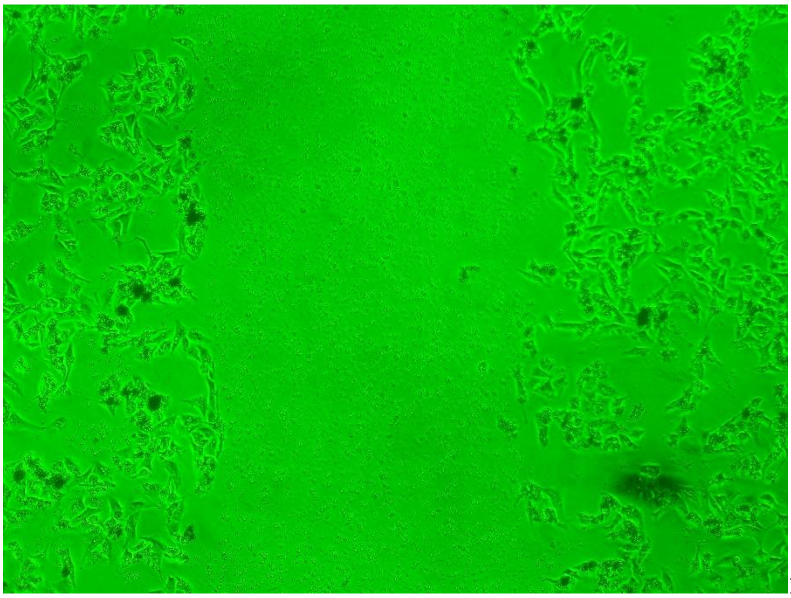
1.24 mM TiO_2 24h

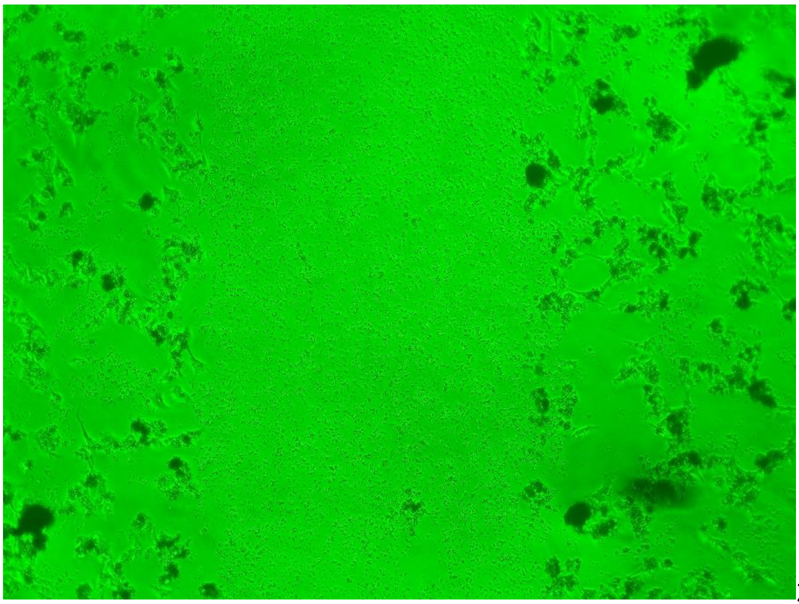
2.54mM TiO_2 24h

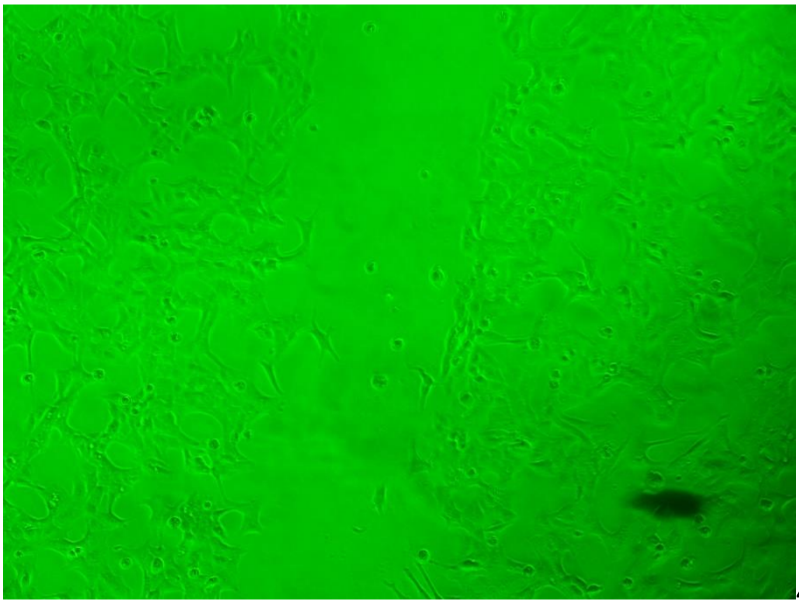
48 hours control

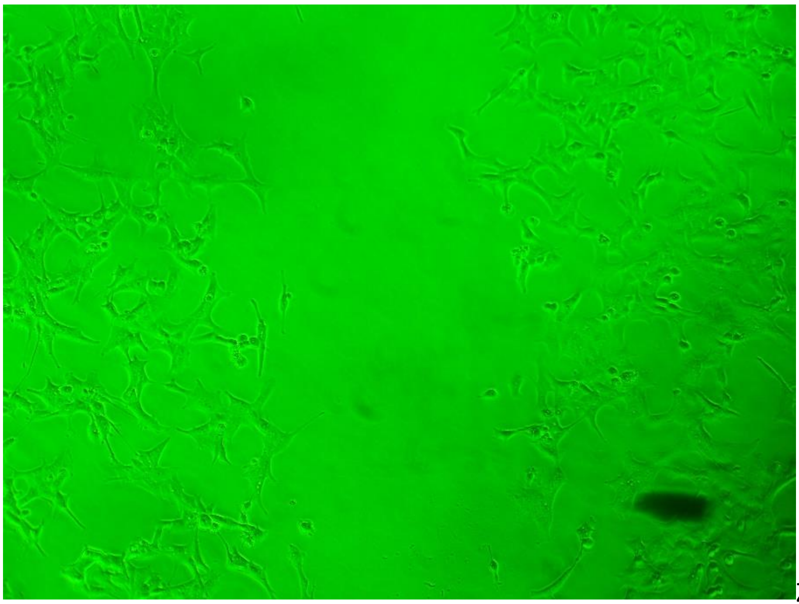
ZnO 0.15mM 48h

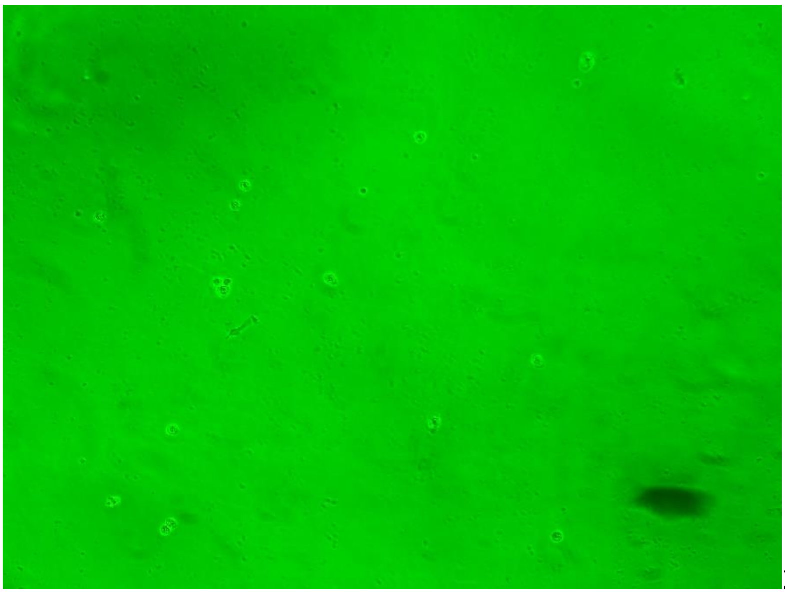
ZnO 0.31mM 48h

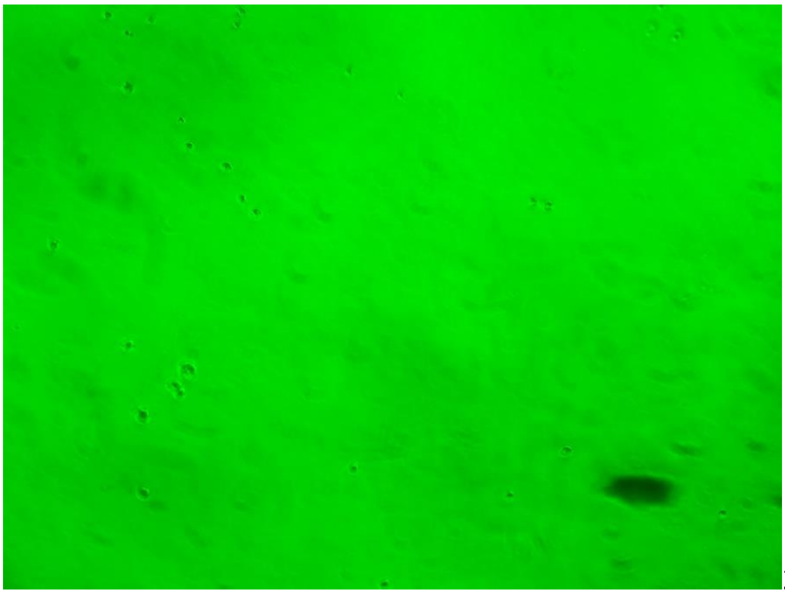
ZnO 0.62mm 48h

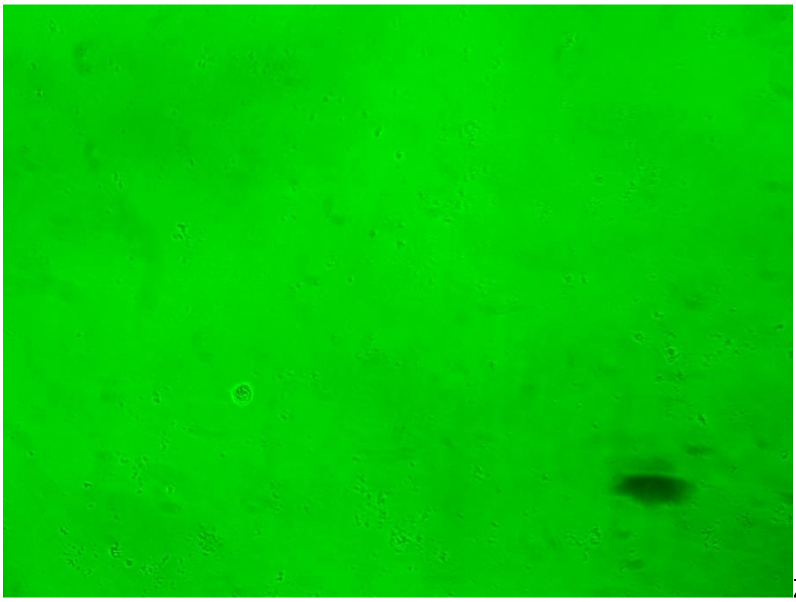
ZnO 1.24mM 48h

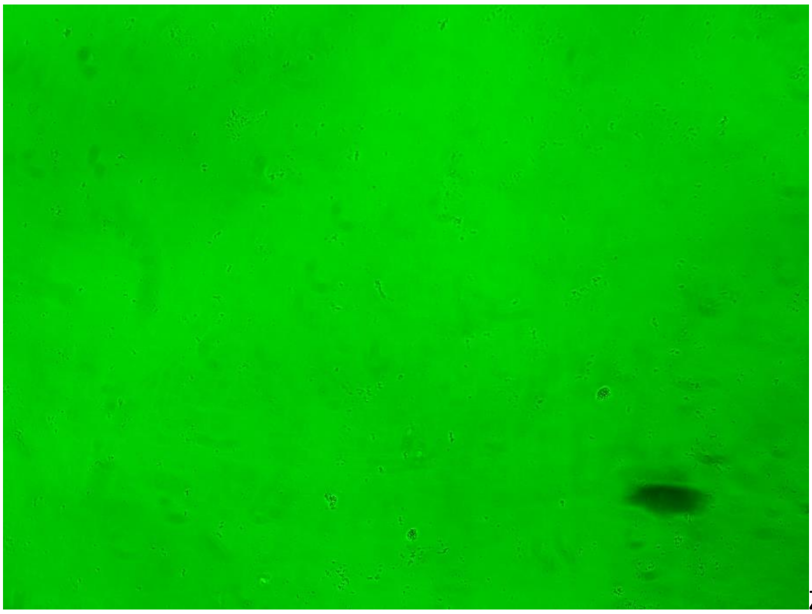
ZnO 2.54mM 48h

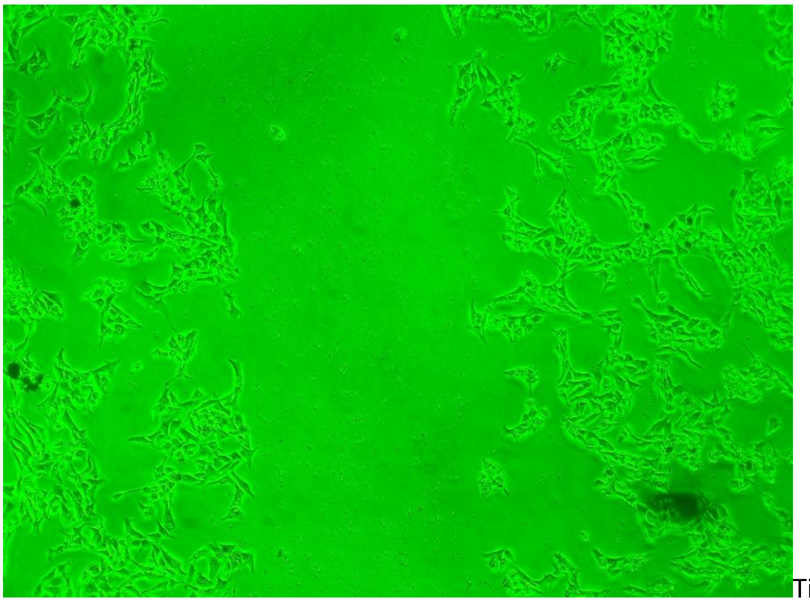
TiO_2 0.15mM 48h

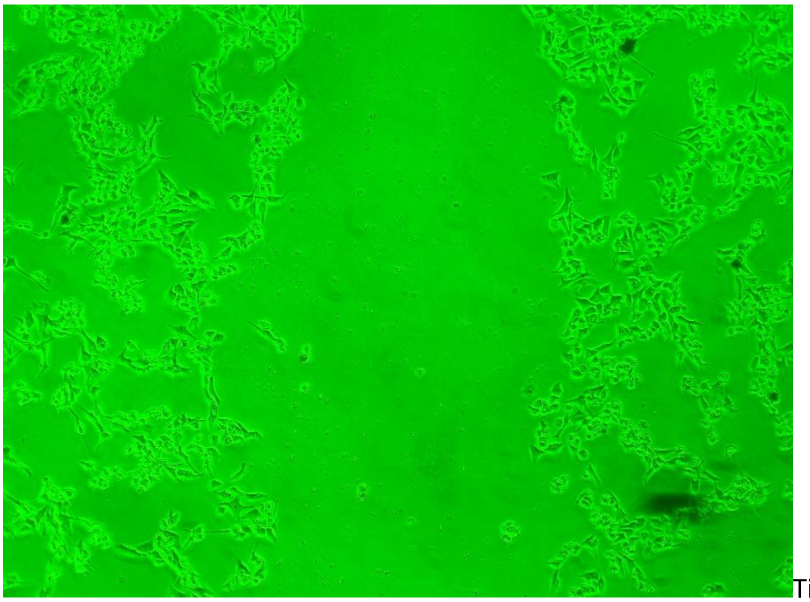
TiO_2 0.31mM 48h

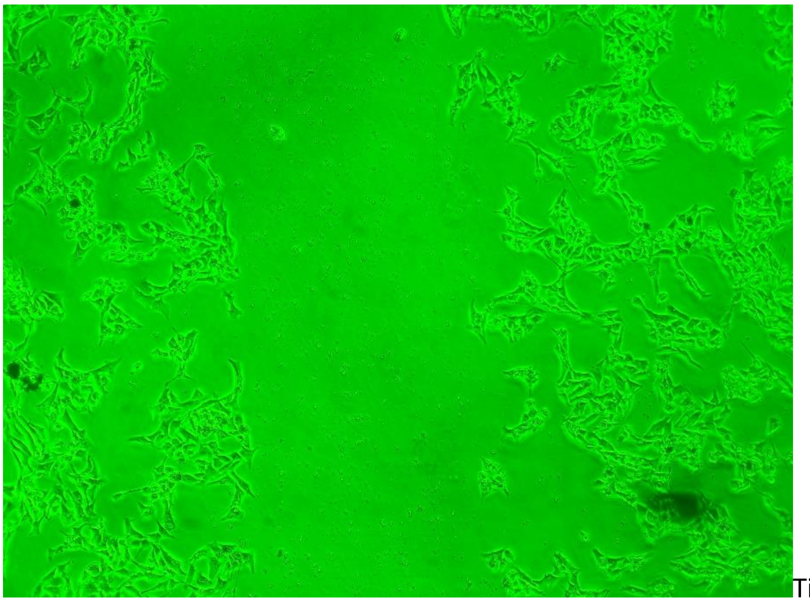
TiO_2 0.62mm 48h

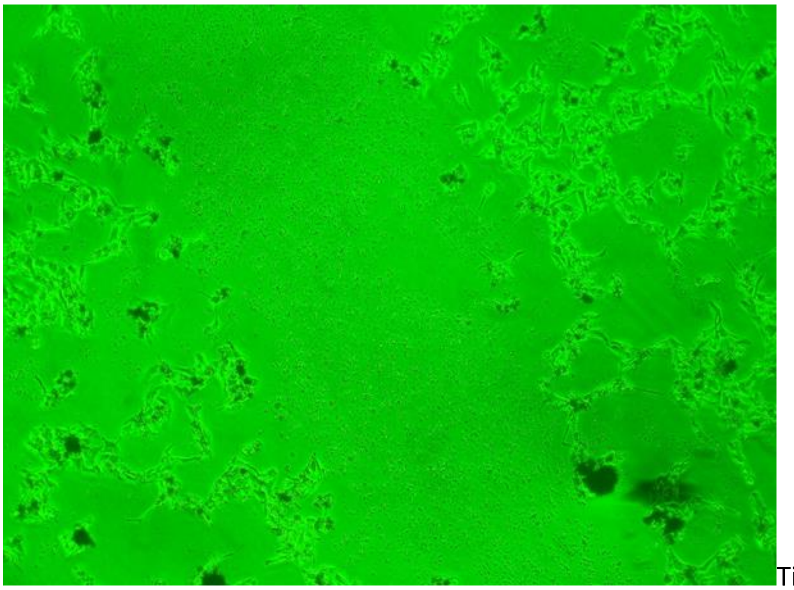
TiO_2 1.24mM 48h

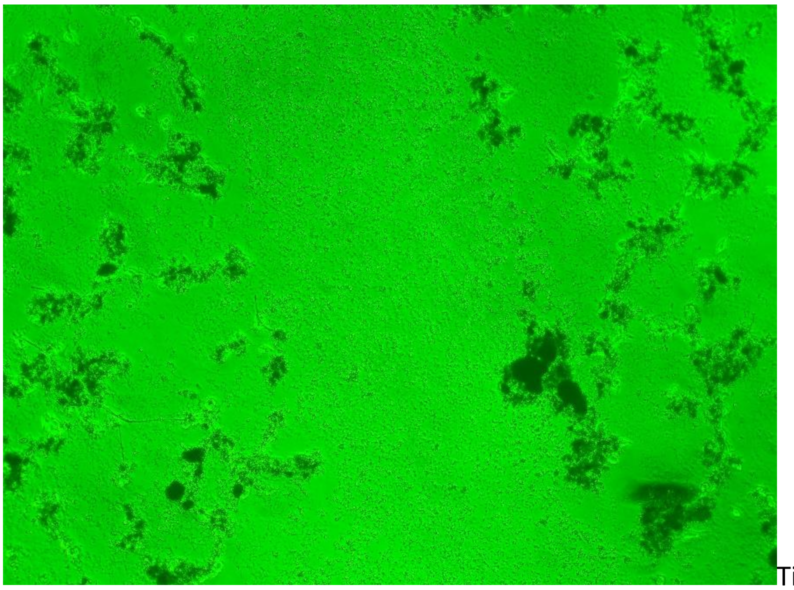
TiO_2 2.54mM 48h

Figure 7 A: **Transwell invasion assay to evaluate migration potential in response to MeOx NP treatment**

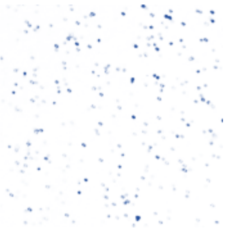
No cells, only membrane

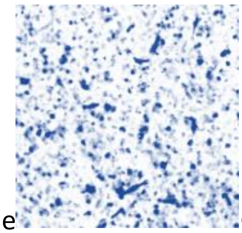
Control 24 hrs

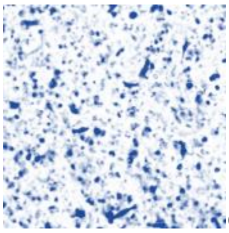
Control 48 hrs

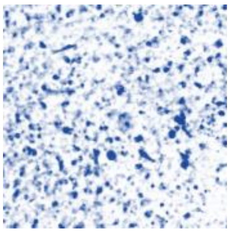
Positive Control

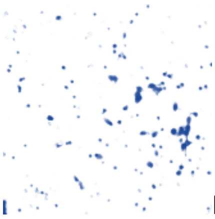
Negative Control

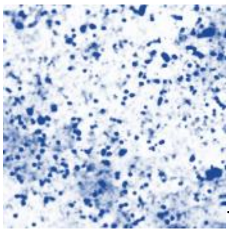
TiO_2 24h 0.15mM

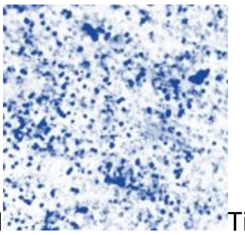
TiO_2 24h 0.31mM

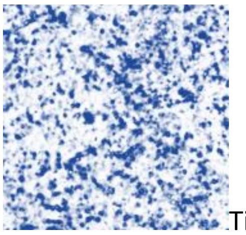
TiO_2 24h 0.62mM

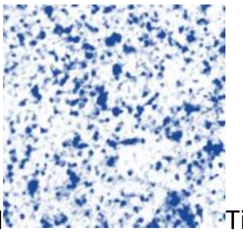
TiO_2 24h 1.24mM

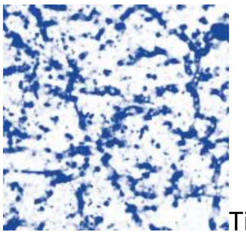
TiO_2 24h 2.54mM

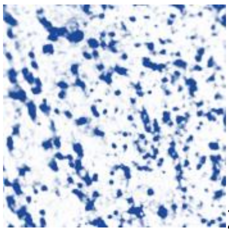
ZnO 0.15mM 24h

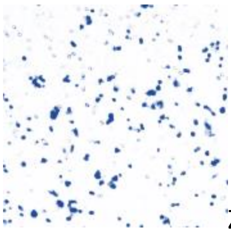
ZnO 0.31mM 24h

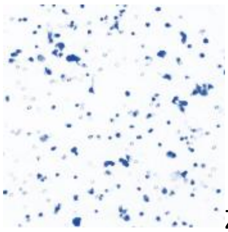
ZnO 0.62mM 24h

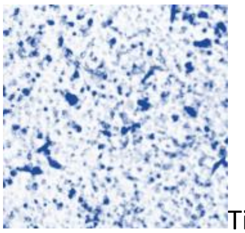
TiO_2 0.15mM 48h

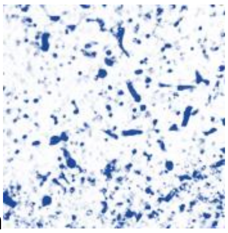
TiO_2 0.31mM 48h

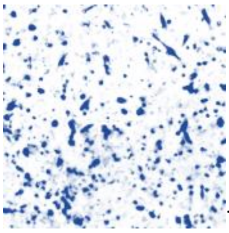
TiO_2 0.62mM 48h

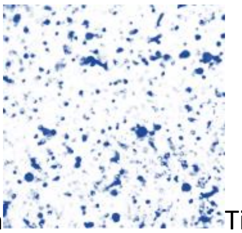
TiO_2 1.24mm 48h

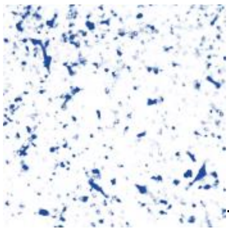
TiO_2 2.54mM 48h

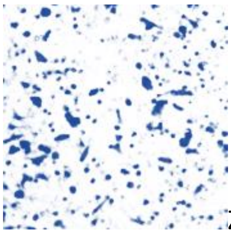
ZnO 0.15mM 48h

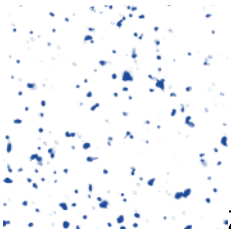
ZnO 0.31mM 48h

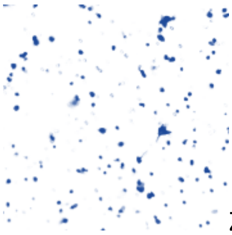
ZnO 0.62mM 48h
